# Public health impacts of increasing the minimum unit price for alcohol in Scotland: A model-based appraisal

**DOI:** 10.1371/journal.pmed.1004792

**Published:** 2026-01-08

**Authors:** John Holmes, Damon Morris, Duncan Gillespie, Alan Brennan, Grace Leeming, Ryan Kai Le Chen, Luke Wilson, Colin Angus

**Affiliations:** Sheffield Addictions Research Group, Sheffield Centre for Health and Related Research, University of Sheffield, Sheffield, United Kingdom; University of Cambridge, UNITED KINGDOM OF GREAT BRITAIN AND NORTHERN IRELAND

## Abstract

**Background:**

Governments in several countries have introduced a minimum unit price (MUP) for alcohol. Evaluation studies suggest this has reduced alcohol-related harm, but MUPs must increase with inflation to remain effective. This paper estimates the impact of the impact of the Scottish Government’s decision to increase its MUP from £0.50 to £0.65 in September 2024 and, alternative options where the MUP changes to between £0.40 and £0.80. It examines impacts on alcohol consumption, spending, and related health outcomes, how impacts vary across the population with regard to deprivation, and how drinkers move between lighter and heavier alcohol consumption groups.

**Methods and findings:**

Policy appraisal using the Sheffield Tobacco and Alcohol Policy Model, a dynamic microsimulation model that combines data on alcohol purchasing and consumption for 10 beverage types and 800 subgroups comprising adults in the Scottish population with price elasticities and an epidemiological model. Deprivation is measured using quintiles of the Scottish Index of Multiple Deprivation. Drinker group is categorised as moderate (<14 units/week, 1 UK unit = 8 g ethanol), hazardous (>14 to ≤35/ ≤50 units/week for women/men), and harmful (>35/50 units/week for women/men). The policy appraisal estimates that, compared to retaining Scotland’s MUP at £0.50, increasing the MUP to £0.65 leads to an estimated 12.0% decrease in alcohol consumption, 2.1% decrease in alcohol spending, 3,385 fewer deaths overall, and 2,578 fewer deaths wholly attributable to alcohol over 20 years. Estimated effects are largest in the quintile of the population living in the most deprived areas. Increasing the MUP to £0.65 is also estimated to reduce the proportion of drinkers consuming at harmful levels by 29.4% and the proportion consuming at hazardous levels by 8.0%. Key limitations of the study include relying on data on alcohol consumption and spending collected before the COVID-19 pandemic, synthesising consumption and spending data from separate datasets, and assuming no supply-side responses (e.g., price changes above the MUP threshold).

**Conclusions:**

Increasing the threshold of an established MUP can lead to additional reductions in alcohol consumption, related harm, and health inequalities. Benefits accrue particularly to the most deprived and heaviest drinkers.

## Author summary

**Why was this study done?:**

- Minimum unit pricing (MUP) sets a floor price for a unit (10 ml or 8 g) of alcohol below which retailers cannot sell alcohol to consumers.
- It is important to increase the floor price over time to prevent inflation, making it less effective, but no study has examined the impact of changes in existing MUP policies.
- The Scottish Government introduced MUP at £0.50 per unit in May 2018 and then raised this to £0.65 per unit in September 2024.

**What did the researchers do and find?:**

- We used the Sheffield Tobacco and Alcohol Policy Model to estimate the impact of increasing Scotland’s MUP threshold to £0.65 per unit and compared this to alternative policy options.
- The model estimates that increasing the MUP threshold to £0.65 could lead to reductions in alcohol consumption, harmful drinking, and deaths or hospitalisation caused by alcohol. Reducing the threshold would lead to increases in these outcomes.
- The estimated effects are largest among heavier drinkers and those living in deprived areas.

**What do these findings mean?:**

- Increasing the MUP threshold can lead to additional reductions in the harm caused by alcohol beyond those arising from introducing the policy.
- MUP policies will become less effective if the price threshold is not increased over time and this will lead to increases in alcohol-related harm.
- Governments that are considering introducing MUP should also consider including automatic increases in the MUP within the relevant legislation.
- Key limitations of this study include relying on data on alcohol consumption from before the COVID-19 pandemic, merging data on alcohol consumption and spending from different sources, and assuming that alcohol producers and retailers did not adjust their business strategies in response to MUP.

## Abstract 3

In this modeling paper, John Holmes and colleagues analyzed the effects on public health of changing the minimum unit price of alcohol in Scotland.

## Introduction

The Scottish Government introduced minimum unit pricing (MUP) for alcohol on 1st May 2018. This prevented the sale of alcohol to consumers for less than £0.50 per unit (1 UK unit = 8 g/10 ml pure alcohol). Inflation since 2018 means the threshold would need to be £0.63 by the end of 2024 to retain its real-terms value [1]. The Scottish Government therefore increased the MUP to £0.65 on 30th September 2024. This paper examines the potential public health impact of changing Scotland’s MUP under a range of scenarios.

Increasing the price of alcohol is among the most effective and cost-effectiveness policy options for reducing alcohol consumption and related harms [2]. MUP is a particularly well-targeted pricing policy as it imposes the largest price increases on the cheapest and highest strength products that are purchased disproportionately by those at greatest risk from alcohol, namely, heavier drinkers on lower incomes [3]. Evaluations of MUP in Scotland suggest the policy reduced alcohol sales by between 3.0% and 3.5% and wholly alcohol-attributable deaths by 13.4% all else being equal [4–7]. Several studies suggest the largest reductions in consumption occurred among heavier drinkers [8–12], but there is some uncertainty as to whether this extended to those drinking at the highest levels, including those with alcohol dependence [13]. However, this appears likely given the evidence of a rapid reduction in deaths from alcohol dependence syndrome and alcohol-related liver disease, which could only arise from reduced drinking among those already experiencing these conditions [7]. Evidence from other jurisdictions with MUP, including Wales and Australia’s Northern Territory, points to similar conclusions regarding the effectiveness of MUP in reducing the public health impact of alcohol [9,14].

MUP thresholds must be increased over time to maintain their effectiveness. Otherwise, rising incomes and price increases caused by inflation mean fewer products will be affected by the policy and those that are will be increasingly affordable. This has been seen previously with alcohol taxes in the US, which have declined in real-terms value by approximately 70% since the 1930s [15]. In contrast, increases in tax rates consistently lead to reduced alcohol consumption and related harm [16,17]. Although there is no direct evidence on the effect of increasing the threshold for MUP policies that are already in place, related research suggests the effect is similar to that of tax increases. Evaluations in the Canadian provinces of British Columbia and Saskatchewan found that increases to the thresholds for less stringent minimum price policies (see [18]) were associated with reductions in alcohol sales, deaths, and hospital admissions, with the largest reductions in admissions occurring among those in more deprived areas [19–22]. The present authors also modelled the effect of setting the initial Scottish MUP at different thresholds prior to its introduction and found higher thresholds led to larger reductions in consumption and alcohol-related harms [23]. However, higher thresholds also had larger effects on moderate drinkers, which may concern policymakers. More evidence is therefore needed on how increasing the MUP in Scotland would affect different groups in the population, including those consuming alcohol at different levels or living in different degrees of deprivation.

In response, the Scottish Government commissioned the authors to conduct a series of analyses to inform its decision-making on the future level of MUP [1]. This paper reports on one of those analyses. It uses a model-based policy appraisal tool, the Sheffield Tobacco and Alcohol Policy Model (STAPM) to estimate the impact of changing Scotland’s MUP from £0.50 per unit to thresholds between £0.40 and £0.80, or removing it entirely. It examines impacts on alcohol consumption, spending, and health outcomes, how impacts vary by deprivation, and how drinkers move between lighter and heavier consumption groups in response to policy changes.

## Methods

### Model overview

**Fig 1:**
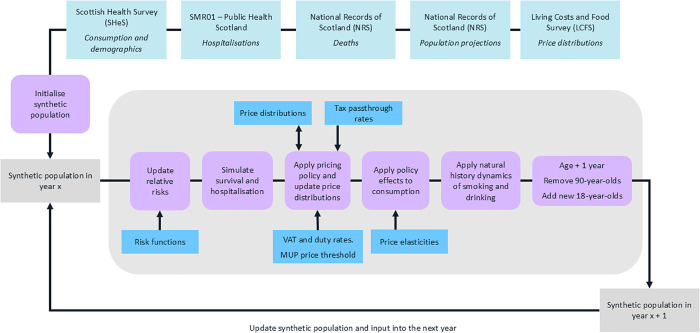
Schematic of the Sheffield Tobacco and Alcohol Policy Model.

The STAPM (https://stapm.gitlab.io) is a dynamic microsimulation modelling tool. It combines econometric, epidemiological, and health economic components to enable detailed analyses of alcohol pricing policy changes. It is similar to our previous Sheffield Alcohol Policy Model [24,25] in its overall structure, functionality, and methodological approach, but differs in two key respects. First, it takes a dynamic approach that allows the characteristics and behaviours of modelled individuals to change over time. This allows us to report changes in the proportion of drinkers within lighter and heavier consumption, which was not previously possible. The dynamic approach also allows us to capture underlying time-trends (e.g., in alcohol consumption) that are unrelated to any modelled policies. We assume that as modelled individuals age, their alcohol consumption follows the age patterns for their population subgroups (see below for data on the consumption data and subgroups). This approach has limitations as it conflates age and cohort trends, but attempting to project forwards the current complex trends in alcohol consumption across age groups would introduce substantial uncertainty into the model [26]. Second, it can incorporate data and policy appraisals related to both tobacco and alcohol, and capture the interacting behavioural and epidemiological effects of these substances. The present analysis only uses this functionality to account for the impact of underlying tobacco consumption trends on health outcomes as other interactions are not relevant. Full methodological details of STAPM, including data sources, are available elsewhere [27,28]. We summarise the methods below, set out a basic model schematic in Fig 1, and provide additional information in the supplementary appendix (S1 Appendix).

### Modelled policies

Scotland introduced MUP on 1st May 2018, with the threshold set at £0.50 per unit. We model nine policy changes: removing the MUP, reducing the threshold to £0.40 and £0.45, and increasing the threshold to £0.55, £0.60. £0.65, £0.70, £0.75, and £0.80. The model works on annual cycles and, in each case, we assume implementation of the new policy in 2019 (i.e., 1 year after the initial MUP) as this is the last year for which we have data unaffected by the COVID-19 pandemic. This is comparable to our previous MUP modelling where we assumed implementation in the last year for which we had data [23]. In all scenarios, we assume that the MUP threshold rises each year from 2020 onwards in line with the Consumer Prices Index including owner occupiers’ housing costs (CPIH) inflation index (i.e., remains constant in real terms; although at the time of writing the Scottish Government has only raised the MUP once, in September 2024). We also assume no changes in household incomes that would affect the affordability of alcohol.

STAPM compares each policy change (or intervention) scenario against a control scenario where the £0.50 MUP remains unchanged in real terms over time. In both the intervention and control scenarios, we initialise the model in 2017, implement the £0.50 MUP in 2018, and then run the simulation until 2040. The intervention scenario thus deviates from the control scenario from 2019 onwards following the policy change.

### Policy to price modelling

The Living Costs and Food Survey (LCFS) is a two-week purchasing diary survey and provides data from 2006 to the first quarter of 2019 on alcohol purchases in the off-trade (i.e., shops) and on-trade (e.g., pubs, restaurants) by individuals within a nationally-representative sample of households resident in Scotland. Prices derived from the Quarter 1 of 2006 to Quarter 1 of 2019 LCFS are calibrated to match observed sales data for Scotland in 2017 [29]. We then construct 8,000 separate price paid distributions describing volumes purchased across price-per-unit of alcohol points for 10 beverage categories (beer, cider, wine, spirits and RTDs [ready-to-drinks or pre-mixed spirits] purchased in the off-trade and on-trade) and 800 population subgroups defined by age (18–24, 25–34, 35–49, 50+), sex, quintiles of equivalised household income, five smoking groups (non-smoker, >0–10 cigarettes/day, >10–20 cigarettes/day, >20–30 cigarettes/day, >30 cigarettes/day) and four drinker group (abstainer; moderate <14 units per week; increasing risk >14 and ≤35 units/week for women or ≤50 units/week for men; high risk >35 units/week for women, >50 units/week for men). From 2018 onwards, all alcohol prices are assumed to rise in line with the Retail Price Index (RPI) inflation index each year to align with UK Government assumptions on alcohol taxes.

To model the introduction of MUP in 2018, we raise the price-per-unit of all purchases below the £0.50 threshold up to the threshold. We use the same approach to model increases in the threshold in 2019. To model the removal or decrease of the threshold, we compare the original 2019 price distribution to the modelled 2019 distribution after the introduction of MUP and decrease the price-per-unit of purchases now unaffected by MUP to their original 2019 level.

### Price to consumption

STAPM models a synthetic population of 200,000 individuals (see [28] for methods) based on the 2016–2018 Scottish Health Survey (SHeS), a nationally-representative survey of individuals resident in Scotland. Respondents to SHeS report their typical weekly alcohol consumption by beverage type via quantity-frequency questions [30]. SHeS provides baseline consumption levels for individuals within the 800 population subgroups. However, it does not separate consumption into off-trade and on-trade, and combines beer and cider into a single category. We therefore separated reported consumption into the 10 beverage categories by using the distribution of purchasing in the LCFS to apportion the consumption reported in SHeS.

Price elasticities describe the effect of a price change in one product on consumption of the same product (own-price) or another product (cross-price). STAPM uses two sets of previously published own- and cross-price elasticities for the 10 beverage types and two tobacco types (cigarettes and roll-your-own) [31]. The first set describes effects of price on participation in consumption and the second set describes effects of price on level of consumption, conditional on participation. We set the tobacco elasticities to zero for the present analysis as estimating the effect of MUP on smoking is out of scope. To model the effect of price changes on alcohol consumption, we calculate the average percentage change in price for each beverage category in each subgroup following the intervention and then apply the price elasticities to estimate changes in participation and consumption level. STAPM then applies this change in consumption to each individual within each subgroup. Alongside the price distributions, this also allows calculation of changes in spending.

Therefore, in effect, our modelling approach means the 800 subgroups each experience a different set of price changes, elasticities, and consumption changes depending on their beverage preferences and prices paid. Unlike in our previous modelling, changes in consumption in STAPM may cause individuals to move between drinker groups and thus population subgroups. We report on these movements in the results.

### Consumption to harm

STAPM uses risk functions to model the impact of consumption changes on levels of mortality and hospitalisation for 45 conditions. We separate these conditions into four categories depending on whether they are wholly or partially attributable to alcohol, and whether they are associated with single drinking occasions (acute conditions) or long-term consumption (chronic conditions). Relative risk functions come from prior literature for partially-attributable chronic conditions [32], mainly the most recent and robust systematic reviews and meta-analyses of the relationship between alcohol consumption and risk of mortality or morbidity for specific conditions. We fit absolute risk functions for wholly-attributable acute and chronic conditions. To model partially-attributable acute conditions, we convert weekly alcohol consumption to estimated patterns of single occasion drinking, then estimate annualised relative risks for each condition (see [28] for methods).

Baseline data on mortality and hospitalisations stratified by age, sex and Scottish Index of Multiple Deprivation (SIMD) quintile were provided to the research team by National Records Scotland and Public Health Scotland. In each year, STAPM assigns modelled individuals a relative risk of death or hospitalisation for each condition based on their current alcohol consumption and lagged effects of consumption in previous years [33]. It then applies the Potential Impact Fraction method to update mortality and morbidity rates [34]. The model uses probabilities of death for each individual based on their subgroup membership and alcohol consumption to simulate who dies in each year and removes those who do from the population.

We report changes in overall and wholly alcohol-attributable deaths, and overall hospitalisations and years of life lost (YLLs) over the modelled period to account for time lags between changes in alcohol consumption and health outcomes [33]. We also report changes in deaths by condition (e.g., cancers, liver disease).

### Sensitivity analyses

We compared results across four sensitivity analyses (SA). First, we compare the impact of using alternative inflation indices by assuming that alcohol prices rise in line with the CPIH, rather than RPI, inflation index. This means we inflate both the MUP threshold and alcohol prices using the same index and ensures all prices remain constant in real terms after 2019. Second, we replace the price elasticities estimated recently by Pryce and colleagues [31] with earlier estimates used in our previous modelling of MUP [35]. Third, we account for SHeS underestimating alcohol consumption relative to more robust sales data by increasing baseline alcohol consumption levels to 80% of total alcohol sales, in line with methods used by the Global Burden of Disease study [36]. Fourth, we examine the impact of accounting for increasing evidence that the association between low levels of drinking and reduced risk for some chronic health conditions, particularly cardiovascular diseases, is a methodological artefact [37,38]. Specifically, we assume that all relative risks below 1.0 in the base case model are instead equal to 1.0.

## Results

### Baseline alcohol consumption and spending

Table 1 shows the baseline alcohol consumption and spending data by SIMD quintile (i.e., the modelled behaviour of the synthetic population in 2019 if MUP remains unchanged at 50p per unit). The abstention rate was 12.1% in the least deprived quintile, approximately half the rate of 23.9% in the most deprived quintile. Mean weekly alcohol consumption and spending was highest in the least deprived quintile. However, although spending declined with increasing deprivation, consumption was similar across the lower four deprivation quintiles.

**Table 1:** Modelled impact of changes to Scotland’s minimum unit price (MUP) threshold on alcohol consumption and spending by Scottish Index of Multiple Deprivation quintile.

	Overall	Quintile 1 (least deprived)	Quintile 2	Quintile 3	Quintile 4	Quintile 5 (most deprived)
Abstainers (%)	17.4%	12.1%	14.2%	17.5%	20.2%	23.9%
Proportion of drinkers	100.0%	23.0%	22.0%	20.0%	19.0%	17.0%
Baseline consumption (units per drinker per week)	12.0	13.6	11.4	12.1	11.2	11.5
Change in consumption by MUP level (%)						
Removed	5.1%	4.5%	4.3%	5.1%	5.7%	6.4%
£0.40	3.3%	2.8%	2.8%	3.3%	3.6%	4.1%
£0.45	2.1%	1.8%	1.8%	2.1%	2.4%	2.7%
£0.50 (unchanged)	0.0%	0.0%	0.0%	0.0%	0.0%	0.0%
£0.55	−2.8%	−2.5%	−2.4%	−2.8%	−3.2%	−3.5%
£0.60	−7.1%	−6.3%	−6.2%	−7.2%	−7.9%	−8.8%
£0.65	−12.0%	−10.7%	−10.3%	−12.2%	−13.3%	−14.7%
£0.70	−18.0%	−16.1%	−15.5%	−18.2%	−20.0%	−22.2%
£0.75	−24.8%	−22.1%	−21.3%	−25.2%	−27.6%	−30.3%
£0.80	−32.7%	−29.1%	−28.0%	−33.2%	−36.5%	−40.4%
Baseline spending (£ per drinker per week)	£27.93	£31.53	£28.07	£27.42	£25.90	£25.80
Change in spending by MUP level (£ per week)						
Removed	0.6%	0.4%	0.4%	0.6%	0.7%	0.9%
£0.40	0.4%	0.3%	0.3%	0.4%	0.5%	0.6%
£0.45	0.2%	0.1%	0.1%	0.2%	0.3%	0.3%
£0.50 (unchanged)	0.0%	0.0%	0.0%	0.0%	0.0%	0.0%
£0.55	−0.4%	−0.4%	−0.3%	−0.4%	−0.5%	−0.6%
£0.60	−1.1%	−1.0%	−0.9%	−1.2%	−1.3%	−1.6%
£0.65	−2.1%	−1.8%	−1.5%	−2.1%	−2.4%	−2.8%
£0.70	−3.3%	−2.8%	−2.5%	−3.4%	−3.9%	−4.6%
£0.75	−4.9%	−4.1%	−3.8%	−5.0%	−5.7%	−6.5%
£0.80	−6.7%	−5.7%	−5.2%	−6.9%	−7.9%	−9.1%

**Fig 2:**
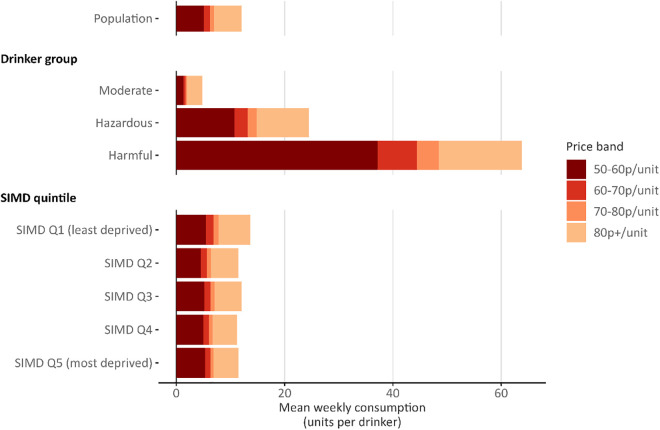
Baseline alcohol consumption by price paid per unit and population subgroup (total length of bar after each colour increment indicates total number of units bought below minimum unit pricing threshold).

Table 2 and Fig 2 show the baseline levels of alcohol consumption at different price-per-unit points under Scotland’s £0.50 MUP by drinker group and SIMD quintile. There were few differences in purchasing patterns across SIMD quintiles. The least deprived quintile purchased 40.1% of their units for less than £0.60 and 50.2% for less than £0.70 whereas the most deprived quintile purchased 46.3% of their units for less than £0.60 and 54.9% for less than £0.70 (proportions below £0.65 are unavailable as the price distributions are calculated at £0.10 intervals). There were greater differences across drinker groups. Moderate drinkers purchased 27.8% of their units for less than £0.60 compared to 44.0% for hazardous drinkers and 58.2% for harmful drinkers.

**Table 2:** Baseline alcohol consumption by price paid per unit and population subgroup.

		Units bought within price band			
	Baseline consumption (units per drinker per week)	50–60p/unit	60–70p/unit	70–80p/unit	80p+/unit
Population	12.0	5.1	1.1	0.8	5.0
Drinker group					
Abstainer	–	–	–	–	–
Moderate^1^	4.8	1.3	0.4	0.3	2.9
Hazardous^2^	24.5	10.8	2.4	1.6	9.6
Harmful^3^	63.9	37.2	7.3	4.0	15.4
SIMD quintile^4^					
Quintile 1 (least deprived)	13.6	5.5	1.4	0.9	5.8
Quintile 2	11.4	4.6	1.1	0.8	5.0
Quintile 3	12.1	5.2	1.2	0.8	5.0
Quintile 4	11.2	5.0	1.0	0.6	4.6
uintile 5 (most deprived)	11.5	5.3	1.0	0.6	4.6

^1^Moderate: Consumes <14 units per week.

^2^Hazardous: Consumes >14 units and ≤35 units per week for women; and >14 units and ≤50 units per week for men.

^3^Harmful: Consumes >35 units per week for women and >50 units per week for men.

^4^SIMD: Scottish Index of Multiple Deprivation.

### Policy appraisal results for consumption and spending

Table 1 also shows the results of the policy appraisals for consumption and spending outcomes. Removing the MUP would lead to an estimated 5.1% increase in alcohol consumption compared to retaining it at £0.50. Changing the MUP from £0.50 to thresholds between £0.40 and £0.80 would lead to estimated changes in alcohol consumption between a 3.3% increase and a 32.7% decrease, with a £0.65 threshold leading to a decrease of 12.0%. All increases in the MUP would lead to reductions in consumption across all deprivation quintiles, but the reductions are largest among the most deprived quintiles.

Removing the MUP would lead to an estimated 0.6% increase in spending on alcohol, while changing the MUP leads to changes in alcohol spending between a 0.4% increase for a £0.40 threshold and a 6.7% decrease for a £0.80 threshold. In each policy scenario, the direction of changes in spending are consistent across SIMD quintiles, but increases and decreases are always larger in the most deprived quintile.

Table 3 shows how the consumption changes lead to changes in the distribution of drinkers across the moderate, hazardous, and harmful drinker groups. Increasing the MUP threshold to £0.65 leads to an estimated 29.4% fewer drinkers consuming at harmful levels, 8.0% fewer drinkers consuming at hazardous levels, and 4.4% more drinkers consuming at moderate levels.

**Table 3:** Modelled impact of changes to Scotland’s minimum unit price (MUP) threshold on the distribution of drinkers across consumption groups.

	Moderate^1^	Hazardous^2^	Harmful^3^
Proportion of drinkers	71.4%	24.6%	4.0%
Number of drinkers in the Scottish population	2,546,501	877,913	143,383
Change in number of drinkers by MUP level (%)			
Removed	−1.5%	1.2%	18.7%
£0.40	−1.0%	0.9%	11.4%
£0.45	−0.6%	0.6%	6.9%
£0.50 (unchanged)	0.0%	0.0%	0.0%
£0.55	1.1%	−1.8%	−7.9%
£0.60	2.7%	−4.8%	−18.5%
£0.65	4.4%	−8.0%	−29.4%
£0.70	6.4%	−12.4%	−38.4%
£0.75	8.6%	−17.0%	−48.8%
£0.80	11.0%	−22.4%	−57.9%

^1^Moderate: Consumes <14 units per week.

^2^Hazardous: Consumes >14 units and ≤35 units per week for women; and >14 units and ≤50 units per week for men.

^3^Harmful: Consumes >35 units per week for women and >50 units per week for men.

### Policy appraisal results for health outcomes

Table 4 shows the number of overall deaths and wholly alcohol-attributable specific deaths in the no change and different MUP scenarios over the modelled period to 2040. Removing the £0.50 MUP would lead to an estimated 1,803 more deaths and 1,206 more wholly alcohol-attributable deaths over this period. In contrast, raising the MUP threshold to £0.65 leads to an estimated 3,385 fewer deaths and 2,578 fewer wholly alcohol-attributable deaths. In the no change scenario, the most deprived quintile had 1.3 times more deaths overall and 3.2 times more wholly alcohol-attributable deaths over the modelled period compared to the least deprived quintile. These inequalities widen when the MUP is removed or decreased and narrow when it is increased. This is because the impact of the policy on overall and wholly alcohol-attributable deaths arising is always estimated to be larger in more deprived groups. For example, when raising the MUP to £0.65, the most deprived quintile (i.e., 20% of the population) accounts for 34% of the reduction in wholly alcohol-attributable deaths.

**Table 4:** Modelled impact of changes to Scotland’s minimum unit price (MUP) threshold on the number of overall and wholly alcohol-attributable deaths over 20-year modelled period by Scottish Index of Multiple Deprivation quintile.

	Overall	Quintile 1 (least deprived)	Quintile 2	Quintile 3	Quintile 4	Quintile 5 (most deprived)
***Overall number of deaths over 20-year modelled period***						
Control scenario	1,115,192	185,211	215,031	230,874	235,919	248,157
Percentage within quintile	100.0%	16.6%	19.3%	20.7%	21.2%	22.3%
Change in MUP scenarios:						
Removed	1,803	206	223	321	445	608
£0.40	1,235	123	143	238	300	431
£0.45	559	62	65	112	124	196
£0.50 (unchanged)	0	0	0	0	0	0
£0.55	−1,063	−118	−143	−214	−268	−320
£0.60	−2,126	−284	−284	−427	−522	−609
£0.65	−3,385	−442	−464	−661	−843	−975
£0.70	−4,808	−588	−672	−903	−1,190	−1,456
£0.75	−6,340	−784	−892	−1,192	−1,511	−1,961
£0.80	−7,879	−993	−1,080	−1,494	−1,805	−2,506
***Number of wholly alcohol-attributable deaths over 20-year modelled period***						
Control scenario	18,240	1,867	2,530	3,287	4,517	6,041
Percentage within quintile	100.0%	10.2%	13.9%	18.0%	24.8%	33.1%
Change in MUP scenarios:						
Removed	1,206	97	138	214	323	434
£0.40	820	63	90	148	209	309
£0.45	420	33	49	78	104	156
£0.50 (unchanged)	0	0	0	0	0	0
£0.55	−724	−62	−87	−129	−199	−247
£0.60	−1,586	−142	−200	−289	−420	−535
£0.65	−2,578	−234	−328	−473	−672	−871
£0.70	−3,651	−335	−469	−664	−940	−1,243
£0.75	−4,747	−434	−620	−870	−1,219	−1,604
£0.80	−5,880	−540	−767	−1,079	−1,500	−1,994

Table 5 shows the same information and pattern of impact on inequalities for YLLs per 100,000 person-years over the modelled period. Raising the MUP to £0.65 is estimated to reduce overall YLL per 100,000 person-years by 230 in the most deprived quintile compared to 54 in the least deprived quintile. Impacts on death rates and hospitalisations are presented in S1 and S2 Tables and also show similar patterns.

**Table 5:** Modelled impact of changes to Scotland’s minimum unit price (MUP) threshold on overall and wholly alcohol-attributable years of life lost (YLLs) over 20-year modelled period by Scottish Index of Multiple Deprivation quintile.

	Overall	Quintile 1 (least deprived)	Quintile 2	Quintile 3	Quintile 4	Quintile 5 (most deprived)
***Overall YLLs per 100,000 person years over modelled period***						
Control scenario	20,447	12,765	16,813	19,947	24,632	30,171
Change in MUP scenarios:						
Removed	65	26	33	59	92	130
£0.40	45	17	22	42	61	92
£0.45	22	9	11	22	29	41
£0.50 (unchanged)	0	0	0	0	0	0
£0.55	−37	−15	−21	−36	−54	−68
£0.60	−79	−34	−45	−76	−110	−142
£0.65	−127	−54	−74	−121	−177	−230
£0.70	−179	−76	−107	−166	−246	−331
£0.75	−233	−100	−140	−216	−318	−432
£0.80	−291	−125	−172	−272	−389	−545

**Fig 3:**
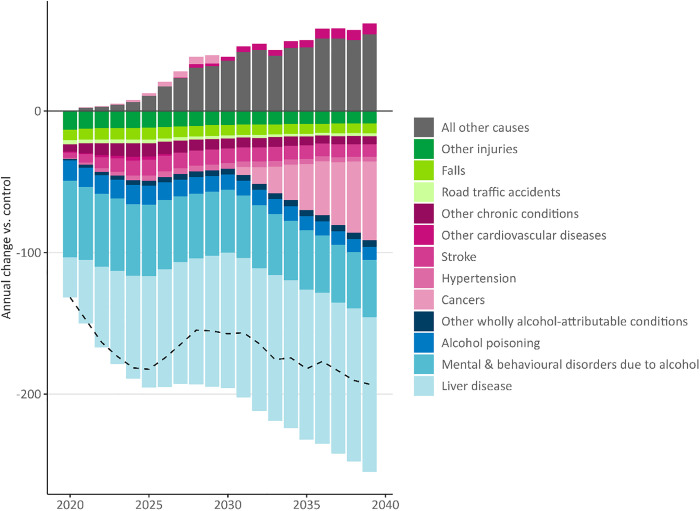
Modelled changes in the annual number of deaths after increasing Scotland’s minimum unit price to £0.65 by year and health condition group (dashed line represents net change in deaths; see S3 Table for numerical data).

Fig 3 shows the estimated annual reduction in deaths from increasing the MUP threshold to £0.65 for different health conditions (see S3 Table for data). Due to the lagged effect of changes in drinking on mortality and variation in this effect across conditions [33], the annual reduction increases from Year 1 to a peak in Year 5, remains stable to Year 10, and then increases each year from Year 11 onwards due to the onset of reductions in deaths from alcohol-attributable cancers. Over the modelled period, liver disease accounts for the largest proportion of the reduction in deaths (51.4%), followed by mental and behavioural disorders due to alcohol (27.1%), and cancers (7.6%). Reductions in deaths from alcohol-attributable conditions are partially offset by increases in deaths from all other causes. Deaths from some cardiovascular diseases also increase as low levels of alcohol consumption may lead to reduced mortality risk [39], although this cardioprotective effect is disputed and increasingly appears likely to be a methodological artefact [37,38].

**Fig 4:**
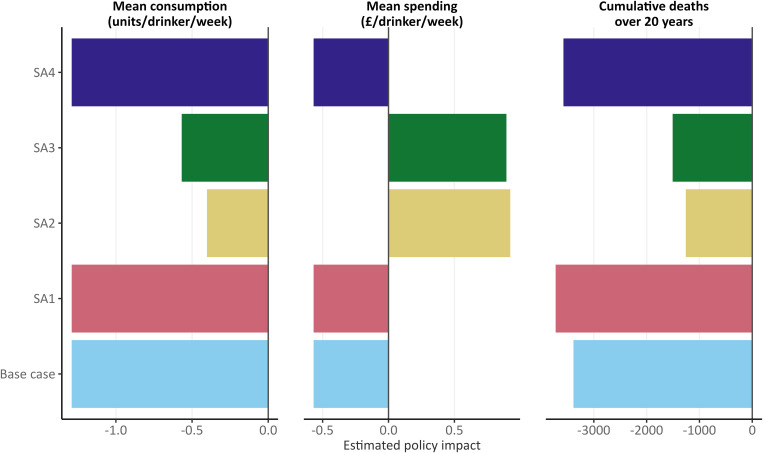
Sensitivity analyses (SA) of modelled changes in alcohol consumption, alcohol spending, and deaths over 20 years for a £0.65 MUP. SA1: Assuming underlying alcohol prices increase in line with CPIH inflation each year, not RPI inflation. SA2: Alternative price elasticities from Meng and colleagues. SA3: Upshifting baseline alcohol consumption to 80% of per capita alcohol sales based on market research data.

Fig 4 shows how the policy appraisal results for increasing the MUP threshold to £0.65 differ between the base case model and the SA (see S4 and S5 Tables for full SA results). Increasing the MUP to £0.65 leads to an estimated reduction in alcohol consumption and overall deaths in all SAs; however, the size of this reduction varies. Assuming underlying prices rise over time by CPIH inflation rather than RPI inflation (SA1) and removing protective effects of moderate drinking (SA4) does not affect the estimated impact of increasing the MUP on alcohol consumption and increases the impact on overall deaths by 10.0% for SA1 and 5.6% for SA4. However, using the alternative elasticity matrix (SA2) and upshifting consumption to account for under-reporting (SA3) reduces the impact on alcohol consumption by 69.2% for SA2 and 53.8% for SA3 and reduces the impact on overall deaths over 20 years by 62.8% for SA2 and 55.5% for SA3. The direction of the impact on alcohol spending also reverses in SA2 and SA3, with average expenditure per drinker per week on alcohol estimated to reduce in these analyses whereas it increases in SA1, SA4, and the base case model.

## Discussion

This study examined the impact of removing or changing Scotland’s £0.50 MUP for alcohol in 2019, with a particular interest in effects in different groups in the population. The policy appraisals found that increasing the MUP threshold led to decreases in alcohol consumption as well as in overall and wholly alcohol-attributable deaths and hospitalisations over 20 years. In contrast, removing or reducing the MUP led to increases for these outcomes. Compared to maintaining the £0.50 threshold, increasing the MUP to the Scottish Government’s chosen level of £0.65 led to an estimated 12.0% reduction in alcohol consumption and 29.4% fewer people drinking at harmful levels. It also led to 3,385 fewer deaths over a 20-year period, including 2,578 fewer wholly alcohol-attributable deaths. These reductions in alcohol consumption and deaths are likely to reduce health inequalities as they are largest among those living in greater deprivation, with around one-third of the reduction in deaths arising in the most deprived quintile of the population. The scale of policy impact varies under SA, and is substantially smaller in some cases. However, the basic pattern of findings remains unchanged except for the impact on alcohol spending, which changes direction and should therefore be considered uncertain.

These results align with previous modelling and evaluation studies examining the impact of alcohol price changes in general and MUP in particular [3,5,7,16,17]. This includes evidence suggesting that alcohol pricing policies can reduce alcohol-attributable health inequalities as they particularly affect heavier drinkers in lower socioeconomic groups [40]. Although the impact of alcohol pricing policies on health conditions linked directly to alcohol, such as alcohol-related liver disease, is well-understood, there is less evidence regarding the impact of pricing policies on rates of cancer mortality [41,42]. Our finding that around 7.6% of the overall reduction in deaths after increasing the MUP arises from reduction in cancer deaths is therefore novel and highlights that a substantial proportion of the impact of alcohol pricing policies relates to conditions that are only partially-attributable to alcohol. It also reflects that an estimated 43.2% of alcohol deaths in the UK and 19.0% internationally are due to cancer [43].

The strengths of this study include that it uses STAPM, a new version of an established alcohol policy modelling tool that permits detailed policy appraisals that account for differences in beverage and pricing preferences across 800 population subgroups. Compared to our previous modelling, the dynamic approach taken in STAPM allows for reporting of new outcomes including changes in how the population is distributed across moderate, hazardous and harmful consumption levels and how health outcomes are distributed across specific conditions.

There are, however, important limitations to the analysis. First, we estimate the impact of removing or changing Scotland’s MUP in 2019 rather than the actual implementation date in 2024. This is because 2019 is the most recent year for which necessary data are available. Projecting trends forward using additional data collected during the COVID-19 pandemic is feasible but would introduce substantial uncertainty. This is because evidence suggests divergent trends in drinking among heavier and lighter drinkers during the pandemic and at the time of analysis there was insufficient evidence on post-pandemic trends [44]. Modelling from 2019 therefore allows greater confidence that modelled impacts are due to MUP rather than other factors but reduces the validity of the findings to the post-pandemic context. Second, and related to the first point, we also do not account for different population subgroups having different trends in alcohol consumption during or after the pandemic. Although there is international evidence of divergent trends in alcohol consumption among lighter and heavier drinkers during the pandemic [44], there is also evidence that trends in alcohol-specific mortality in England were similar across deprivation group [45]. In the absence of post-pandemic data on alcohol consumption in Scotland, imposing assumptions about these complex trends would introduce substantial uncertainty in the model. Third, we had to synthesise individual-level data on alcohol consumption and spending for each beverage category from multiple sources as no single dataset provides complete information. This may have introduced biases where the synthesis inaccurately captured the behaviour of particular subgroups. Fourth, STAPM does not take account of potential secondary responses to changes in the MUP, such as changes in the products available for sale, changes to prices above the MUP threshold, increased treatment-seeking by those with alcohol dependence, or increased illicit and cross-border purchasing. However, Public Health Scotland’s evaluation of the initial MUP found such responses were typically limited in scale and concentrated in small subsections of the population (e.g., those who lived close to the Scotland-England border) [46]. Fifth, STAPM also does not account for the introduction of other policies that may affect the sale of cheap alcohol, including the 2023 reforms to UK alcohol taxes. However, these reforms are unlikely to substantially change the impact of MUP in Scotland as the resulting prices changes were small compared to those imposed by MUP, and concentrated on products largely unaffected by MUP (i.e., wines and RTDs) [47].

At the time of writing, four jurisdictions have a MUP for their whole alcohol market: Armenia, Ireland, Scotland, and Wales (Australia’s Northern Territory repealed its MUP in March 2025 following a change of government [48]). Our findings suggest that increasing the MUP threshold in these jurisdictions would deliver additional health benefits and reductions in health inequalities. They further suggest that failing to routinely increase the MUP to account for inflation will degrade its effectiveness over time as the price threshold will fall in real terms. Although some jurisdictions have included powers that give governments the option to review and change the threshold within legislation, no country has committed to routine annual increases. The reasons for this are unclear, but may include disruption to policymaking during the COVID-19 pandemic, a commitment to waiting for evaluation results from the initial implementation, or changes in government or government priorities. Failing to raise the MUP risks it falling victim to the same problem that afflicts alcohol taxation in some countries, where tax rates decline in real terms and are adjusted only intermittently and to an insufficient degree to maintain their effectiveness [49]. Jurisdictions considering introducing an MUP should therefore consider and agree mechanisms for increases at the point of legislation to ensure measures are in place to maintain policy effectiveness over time. Following roundtable discussions with policy stakeholders, we have previously recommended a mixture of mandating annual increases in line with a preferred measure of inflation and periodic reviews (e.g., every five years) to ensure continued effectiveness [1]. Although such measures would not guarantee annual increases, they would mean that raising the MUP is the default policy and a deviation from this is an active, rather than passive, policy decision.

Analyses and evaluation of MUP suggest it is effective in achieving reductions in alcohol consumption and related harms among those at greatest risk. However, it may be less effective in reducing alcohol consumption among groups that do not buy cheap alcohol [3]. Revenue from the increased prices under MUP also goes to commercial actors and provides no additional income to governments. Combining MUP with additional measures, such as increases in alcohol taxation or investment in early identification and treatment of alcohol use disorders, may therefore deliver additional benefits. Alternatively, combining tax increases with minimum tax thresholds may be feasible in some jurisdictions, although there are practical and political challenges to this approach within the UK tax system [50]. Many regional governments also lack tax-raising powers, and this is among the reasons the Scottish Government pursued MUP in the first place.

While MUP reduces alcohol-related harm, concerns exist about its financial impact on people with alcohol dependence who are also economically vulnerable [51], especially if increases in the price threshold exceed inflation. Political considerations also arise regarding effects on low-risk drinkers or potential reductions in public support for the policy if the price thresholds move too high [52]. Policymakers therefore face trade-offs because a higher MUP would provide larger reductions in harm but potentially more undesirable impacts. Ensuring alcohol treatment services are adequately resourced, prepared, and equipped to support clients experiencing difficulties due to alcohol price increases may help to mitigate harms. Managed Alcohol Programmes or engagement with clients regarding their financial situation may also be relevant interventions [51,53]. Conversely, as noted by alcohol treatment providers and service users in evaluations of MUP in Scotland and Wales [54,55], the policy is likely to prevent people from becoming dependent on alcohol in the first place and, therefore, it will reduce negative impacts through that mechanism as well.

Future research should evaluate any enacted changes to MUP levels in Scotland and other jurisdictions to provide real-world evidence of their impact on alcohol consumption, health, and health inequality outcomes. It should also seek methods to incorporate the complex changes in alcohol consumption and related harms that occurred during the COVID-19 pandemic into dynamic simulation models, including STAPM. The growing number of studies evaluating MUP also affords opportunities to improve policy modelling tools by providing real-world evidence that researchers can use to calibrate such tools. This may include exploring synergistic interactions between two or more alcohol policies introduced at the same time.

In conclusion, increasing the threshold of Scotland’s MUP policy is likely to lead to reductions in alcohol consumption and related harms. The health benefits are likely to be greatest for those in more deprived groups and heavier drinkers.

## Supporting information

S1 Table Modelled impact of changes to Scotland’s minimum unit price (MUP) threshold on the number of alcohol- and tobacco-related hospitalisations over 20-year modelled period by Scottish Index of Multiple Deprivation quintile. (DOCX)

S2 Table Modelled impact of changes to Scotland’s minimum unit price (MUP) threshold on the overall and wholly alcohol-attributable death rate over 20-year modelled period by Scottish Index of Multiple Deprivation quintile. (DOCX)

S3 Table Modelled changes in the annual number of deaths after increasing Scotland’s minimum unit price to £0.65 by year and health condition group. (DOCX)

S4 Table Modelled consumption and spending outcomes for a 65p minimum unit price (MUP) under different Sensitivity Analyses. (DOCX)

S5 Table Modelled health outcomes over 20 years for a 65p minimum unit price (MUP) under different Sensitivity Analyses. (DOCX)

S1 Appendix **Table A1.** Health conditions wholly or partially attributable to alcohol. **Table A2.** Health conditions wholly or partially attributable to tobacco. **Table A3.** Health conditions wholly or partially attributable to tobacco. **Table A4.** Base case Pryce and colleagues elasticities (participation). **Table A5.** Base Case Pryce and colleagues elasticities (conditional consumption). **Table A6.** Meng and colleagues (2014) alcohol price elasticities. **Fig A1.** Schematic of the tobacco and alcohol tax and price policy simulation model. **Fig A2.** Summary of the TAX-sim workflow for calculating price and consumption effects. **Fig A3.** Illustration of the impact of a hypothetical MUP policy on the price distribution.
(DOCX)

## Abbreviations

LCFS: Living Costs and Food Survey
MUP: minimum unit price
SA: sensitivity analyses
SHeS: Scottish Health Survey
SIMD: Scottish Index of Multiple Deprivation
STAPM: Sheffield Tobacco and Alcohol Policy Model
YLLs: years of life lost
