# Supplementary material for: Public health impacts of increasing the minimum unit price for alcohol in Scotland: A model-based appraisal
Source: PLoS Med. 2026 Jan 8;23(1):e1004792. doi: 10.1371/journal.pmed.1004792 (PMC12782643; doi:10.1371/journal.pmed.1004792)
Supplement: S1 Table — (DOCX) [file pmed.1004792.s001.docx]

*Table S1: Modelled impact of changes to Scotland’s minimum unit price (MUP) threshold on the number of alcohol- and tobacco-related hospitalisations over 20-year modelled period by Scottish Index of Multiple Deprivation quintile.*

|  | Overall | Quintile 1  (least deprived) | Quintile 2 | Quintile 3 | Quintile 4 | Quintile 5  (most deprived) |
| --- | --- | --- | --- | --- | --- | --- |
| ***Alcohol- and tobacco-related hospitalisations over modelled period^1^*** |  |  |  |  |  |  |
| Control scenario | 5,543,064 | 924,937 | 978,221 | 1,060,162 | 1,193,250 | 1,386,494 |
| Change in MUP scenarios: |  |  |  |  |  |  |
| Removed | 21,997 | 2,494 | 2,313 | 3,729 | 5,598 | 7,863 |
| £0.40 | 15,166 | 1,603 | 1,554 | 2,630 | 3,774 | 5,606 |
| £0.45 | 7,464 | 826 | 803 | 1,357 | 1,854 | 2,624 |
| £0.50 (unchanged) | 0 | 0 | 0 | 0 | 0 | 0 |
| £0.55 | -13,179 | -1,501 | -1,530 | -2,299 | -3,430 | -4,419 |
| £0.60 | -27,774 | -3,351 | -3,373 | -4,998 | -6,877 | -9,174 |
| £0.65 | -44,922 | -5,439 | -5,551 | -8,044 | -11,072 | -14,817 |
| £0.70 | -63,698 | -7,741 | -8,037 | -11,195 | -15,418 | -21,308 |
| £0.75 | -83,256 | -10,194 | -10,600 | -14,645 | -19,977 | -27,838 |
| £0.80 | -103,894 | -12,814 | -13,179 | -18,334 | -24,502 | -35,066 |

*^1^All hospitalisations for conditions related to alcohol or tobacco use. See appendix Tables 1-3 for list of conditions.*
