# Supplementary material for: Public health impacts of increasing the minimum unit price for alcohol in Scotland: A model-based appraisal
Source: PLoS Med. 2026 Jan 8;23(1):e1004792. doi: 10.1371/journal.pmed.1004792 (PMC12782643; doi:10.1371/journal.pmed.1004792)
Supplement: S2 Table — (DOCX) [file pmed.1004792.s002.docx]

*Table S2: Modelled impact of changes to Scotland’s minimum unit price (MUP) threshold on the overall and wholly alcohol-attributable death rate over 20-year modelled period by Scottish Index of Multiple Deprivation quintile.*

|  | Overall | Quintile 1  (least deprived) | Quintile 2 | Quintile 3 | Quintile 4 | Quintile 5  (most deprived) |
| --- | --- | --- | --- | --- | --- | --- |
| ***Overall deaths per 100,000 person years over modelled period*** |  |  |  |  |  |  |
| Control scenario | 1,310.50 | 971.4 | 1,217.30 | 1,364.60 | 1,507.10 | 1,571.30 |
| Change in MUP scenarios: |  |  |  |  |  |  |
| Removed | 2.1 | 1.1 | 1.3 | 1.9 | 2.8 | 3.9 |
| £0.40 | 1.5 | 0.6 | 0.8 | 1.4 | 1.9 | 2.7 |
| £0.45 | 0.7 | 0.3 | 0.4 | 0.7 | 0.8 | 1.2 |
| £0.50 (unchanged) | 0 | 0 | 0 | 0 | 0 | 0 |
| £0.55 | -1.2 | -0.6 | -0.8 | -1.3 | -1.7 | -2 |
| £0.60 | -2.5 | -1.5 | -1.6 | -2.5 | -3.3 | -3.9 |
| £0.65 | -4.0 | -2.3 | -2.6 | -3.9 | -5.4 | -6.2 |
| £0.70 | -5.6 | -3.1 | -3.8 | -5.3 | -7.6 | -9.2 |
| £0.75 | -7.4 | -4.1 | -5.0 | -7.0 | -9.6 | -12.4 |
| £0.80 | -9.2 | -5.2 | -6.1 | -8.8 | -11.5 | -15.8 |
| ***Wholly alcohol-attributable deaths per 100,000 person years over modelled period*** |  |  |  |  |  |  |
| Control scenario | 21.4 | 9.8 | 14.3 | 19.4 | 28.9 | 38.2 |
| Change in MUP scenarios: |  |  |  |  |  |  |
| Removed | 1.4 | 0.5 | 0.8 | 1.3 | 2.1 | 2.8 |
| £0.40 | 1.0 | 0.3 | 0.5 | 0.9 | 1.3 | 2 |
| £0.45 | 0.5 | 0.2 | 0.3 | 0.5 | 0.7 | 1 |
| £0.50 (unchanged) | 0 | 0 | 0 | 0 | 0 | 0 |
| £0.55 | -0.9 | -0.3 | -0.5 | -0.8 | -1.3 | -1.6 |
| £0.60 | -1.9 | -0.7 | -1.1 | -1.7 | -2.7 | -3.4 |
| £0.65 | -3.0 | -1.2 | -1.9 | -2.8 | -4.3 | -5.5 |
| £0.70 | -4.3 | -1.8 | -2.7 | -3.9 | -6.0 | -7.9 |
| £0.75 | -5.6 | -2.3 | -3.5 | -5.1 | -7.8 | -10.1 |
| £0.80 | -6.9 | -2.8 | -4.3 | -6.4 | -9.6 | -12.6 |
