# Supplementary material for: Public health impacts of increasing the minimum unit price for alcohol in Scotland: A model-based appraisal
Source: PLoS Med. 2026 Jan 8;23(1):e1004792. doi: 10.1371/journal.pmed.1004792 (PMC12782643; doi:10.1371/journal.pmed.1004792)
Supplement: S3 Table — (DOCX) [file pmed.1004792.s003.docx]

*Table S3: Modelled changes in annual number of deaths after increasing Scotland’s minimum unit price to £0.65 by year and health condition group.*

|  | 2020 | 2021 | 2022 | 2023 | 2024 | 2025 | 2026 | 2027 | 2028 | 2029 | 2030 | 2031 | 2032 | 2033 | 2034 | 2035 | 2036 | 2037 | 2038 | 2039 |
| --- | --- | --- | --- | --- | --- | --- | --- | --- | --- | --- | --- | --- | --- | --- | --- | --- | --- | --- | --- | --- |
| All other causes (not alcohol-related) | 0 | 2 | 3 | 4 | 6 | 11 | 17 | 23 | 31 | 32 | 36 | 42 | 43 | 39 | 44 | 45 | 51 | 51 | 50 | 54 |
| Other injuries (not alcohol-related) | -13 | -13 | -12 | -12 | -12 | -12 | -11 | -11 | -10 | -10 | -10 | -10 | -10 | -9 | -9 | -9 | -9 | -9 | -9 | -9 |
| Falls | -7 | -7 | -8 | -8 | -8 | -8 | -8 | -8 | -7 | -7 | -7 | -7 | -7 | -7 | -7 | -7 | -6 | -7 | -7 | -7 |
| Road traffic accidents | -3 | -3 | -3 | -3 | -3 | -3 | -2 | -2 | -2 | -2 | -2 | -2 | -2 | -2 | -2 | -2 | -2 | -2 | -2 | -2 |
| Other chronic conditions | -5 | -7 | -8 | -9 | -10 | -9 | -9 | -8 | -7 | -7 | -7 | -7 | -7 | -7 | -6 | -6 | -6 | -6 | -6 | -6 |
| Other cardiovascular diseases | -1 | -1 | -1 | -2 | -3 | -2 | -1 | 0 | 2 | 2 | 3 | 4 | 5 | 4 | 5 | 5 | 7 | 7 | 7 | 8 |
| Stroke | -3 | -6 | -8 | -9 | -11 | -11 | -11 | -11 | -11 | -11 | -10 | -10 | -10 | -10 | -10 | -10 | -9 | -9 | -9 | -9 |
| Hypertension | -1 | -2 | -2 | -3 | -3 | -4 | -4 | -4 | -4 | -4 | -4 | -4 | -4 | -4 | -4 | -4 | -4 | -4 | -4 | -3 |
| Cancers | 0 | 0 | 1 | 1 | 1 | 2 | 3 | 4 | 5 | 6 | 0 | -6 | -12 | -19 | -25 | -32 | -38 | -44 | -50 | -56 |
| Other wholly alcohol-attributable conditions | -1 | -2 | -3 | -3 | -3 | -4 | -4 | -4 | -4 | -4 | -4 | -4 | -4 | -4 | -4 | -4 | -4 | -4 | -5 | -5 |
| Alcohol poisoning | -14 | -14 | -13 | -13 | -13 | -13 | -13 | -12 | -12 | -11 | -11 | -11 | -11 | -11 | -10 | -10 | -10 | -10 | -9 | -9 |
| Mental & behavioural disorders due to alcohol | -54 | -52 | -52 | -51 | -51 | -50 | -49 | -46 | -46 | -45 | -44 | -44 | -44 | -43 | -42 | -42 | -41 | -41 | -40 | -41 |
| Liver disease | -28 | -45 | -57 | -66 | -73 | -79 | -83 | -86 | -89 | -93 | -95 | -98 | -101 | -103 | -104 | -106 | -107 | -107 | -108 | -109 |
| Total change | -132 | -148 | -163 | -173 | -181 | -182 | -174 | -165 | -155 | -155 | -157 | -157 | -164 | -176 | -174 | -182 | -177 | -184 | -190 | -193 |
