# Supplementary material for: Public health impacts of increasing the minimum unit price for alcohol in Scotland: A model-based appraisal
Source: PLoS Med. 2026 Jan 8;23(1):e1004792. doi: 10.1371/journal.pmed.1004792 (PMC12782643; doi:10.1371/journal.pmed.1004792)
Supplement: S4 Table — (DOCX) [file pmed.1004792.s004.docx]

*Table S4: Modelled consumption and spending outcomes for a 65p MUP under different Sensitivity Analyses*

|  | Base case | SA1 - CPIH price increases | SA2 - Meng elasticities | SA3 - upshifted consumption | SA4 – protective effects removed |
| --- | --- | --- | --- | --- | --- |
| Baseline alcohol consumption (units/drinker/week) | 12.0 | 12.0 | 12.9 | 17.6 | 12.0 |
| Absolute change under 65p MUP vs. control | -1.3 | -1.3 | -0.4 | -0.6 | -1.3 |
| Relative change | -12.0% | -12.0% | -3.2% | -3.3% | -12.0% |
|  |  |  |  |  |  |
| Baseline spending on alcohol (£ per drinker/week) | 27.9 | 27.9 | 28.8 | 28.2 | 27.9 |
| Absolute change under 65p MUP vs. control | -0.6 | -0.6 | 0.9 | 0.9 | -0.6 |
| Relative change | -2.1% | -2.1% | 3.1% | 3.1% | -2.1% |
