# Supplementary material for: Public health impacts of increasing the minimum unit price for alcohol in Scotland: A model-based appraisal
Source: PLoS Med. 2026 Jan 8;23(1):e1004792. doi: 10.1371/journal.pmed.1004792 (PMC12782643; doi:10.1371/journal.pmed.1004792)
Supplement: S5 Table — (DOCX) [file pmed.1004792.s005.docx]

*Table S5: Modelled health outcomes over 20 years for a 65p MUP under different Sensitivity Analyses*

|  | Base case | SA1 - CPIH price increases | SA2 - Meng elasticities | SA3 - upshifted consumption | SA4 – protective effects removed |
| --- | --- | --- | --- | --- | --- |
| Overall number of deaths over 20 year period (control scenario) | 1,115,192 | 1,114,454 | 1,117,795 | 1,119,308 | 1,117,347 |
| Change under 65p MUP | -3,385 | -3,722 | -1,259 | -1,508 | -3,575 |
|  |  |  |  |  |  |
| Overall number of hospital admissions over 20 year period (control scenario) | 5,543,064 | 5,533,320 | 5,570,988 | 5,578,142 | 5,543,090 |
| Change under 65p MUP | -44,922 | -48,455 | -18,213 | -23,279 | -45,554 |
|  |  |  |  |  |  |
| Overall number of Years of Life Lost to premature mortality (control scenario) | 17,399,600 | 17,376,908 | 17,469,839 | 17,484,449 | 17,433,557 |
| Change under 65p MUP | -107,938 | -116,129 | -43,250 | -54,859 | -109,423 |
