# Supplementary material for: Public health impacts of increasing the minimum unit price for alcohol in Scotland: A model-based appraisal
Source: PLoS Med. 2026 Jan 8;23(1):e1004792. doi: 10.1371/journal.pmed.1004792 (PMC12782643; doi:10.1371/journal.pmed.1004792)
Supplement: S1 Appendix — Table A1. Health conditions wholly or partially attributable to alcohol. Table A2. Health conditions wholly or partially attributable to tobacco. Table A3. Health conditions wholly or partially attributable to tobacco. Table A4. Base case Pryce and colleagues elasticities (participation). Table A5. Base Case Pryce and colleagues elasticities (conditional consumption). Table A6. Meng and colleagues (2014) alcohol price elasticities. Fig A1. Schematic of the tobacco and alcohol tax and price policy simulation model. Fig A2. Summary of the TAX-sim workflow for calculating price and consumption effects. Fig A3. Illustration of the impact of a hypothetical MUP policy on the price distribution. (DOCX) [file pmed.1004792.s006.docx]

Public health impacts of increasing the minimum unit price for alcohol in Scotland: A model-based appraisal.

Supplementary Appendix

Table of Contents

[INDEX OF TABLES 2](#_Toc206058588)

[INDEX OF FIGURES 2](#_Toc206058589)

[1 Methodological Appendix 2](#_Toc206058590)

[1.1 Introduction 2](#_Toc206058591)

[1.1.1 Joint Tobacco and Alcohol Policy Modelling 2](#_Toc206058592)

[1.1.2 The STAPM Platform 2](#_Toc206058593)

[1.2 Model Overview 3](#_Toc206058594)

[1.2.1 Description of the model structure 3](#_Toc206058595)

[1.2.2 Initialising the model 5](#_Toc206058596)

[1.2.3 Data sources 6](#_Toc206058597)

[1.3 Microsimulation of alcohol consumption dynamics 14](#_Toc206058598)

[1.3.1 Alcohol consumption 15](#_Toc206058599)

[1.3.2 Participation in alcohol consumption 18](#_Toc206058600)

[1.3.3 Alcohol conditional consumption 19](#_Toc206058601)

[1.3.4 Alcohol product preferences 20](#_Toc206058602)

[1.3.5 Alcohol consumption outcomes 21](#_Toc206058603)

[1.4 Modelling the relationship between consumption and harms 22](#_Toc206058604)

[1.4.1 Relative risks of disease 23](#_Toc206058605)

[1.4.2 Calculating population attributable fractions to estimate the total avoidable burden of mortality and morbidity due to tobacco and alcohol in a single year 27](#_Toc206058606)

[1.4.3 Linking behaviour change to change in mortality and morbidity between years in the microsimulation 27](#_Toc206058607)

[1.4.4 Mortality analysis 28](#_Toc206058608)

[1.4.5 Morbidity analysis 30](#_Toc206058609)

[1.5 Modelling the effects of pricing policy on prices and consumption 31](#_Toc206058610)

[1.5.1 Modelling tax policy to price 32](#_Toc206058611)

[1.5.2 Modelling minimum unit pricing 34](#_Toc206058612)

[1.5.3 Modelling price to consumption 36](#_Toc206058613)

[1.5.4 Updating consumption 40](#_Toc206058614)

[1.5.5 Calculating economic outcomes 41](#_Toc206058615)

# INDEX OF TABLES

[Table A1 Health conditions wholly or partially attributable to alcohol 13](#_Toc184804582)

[Table A2 Health conditions wholly or partially attributable to tobacco 14](#_Toc184804583)

[Table A3 Health conditions wholly or partially attributable to tobacco 15](#_Toc184804584)

[Table A5 Base Case Pryce et al. elasticities (participation) 42](#_Toc184804585)

[Table A5 Base Case Pryce et al. elasticities (conditional consumption) 42](#_Toc184804586)

[Table A6 Meng et al. (2014) alcohol price elasticities 43](#_Toc184804587)

# INDEX OF FIGURES

[Figure A1 Schematic of the Tobacco and Alcohol Tax and Price Policy Simulation Model 6](#_Toc184805471)

[Figure A2 Summary of the TAX-Sim workflow for calculating price and consumption effects. 32](#_Toc184805472)

[Figure A3 Illustration of the impact of a hypothetical MUP policy on the price distribution. 37](#_Toc184805473)

# Methodological Appendix

## Introduction

This methodological appendix provides a detailed technical explanation of the Sheffield Tobacco and Alcohol Policy Modelling (STAPM). STAPM was developed in R and R studio. The data and code in the STAPM platform can be used to construct different types of models. The Tobacco and Alcohol Tax and Price Intervention Simulation Model (TAX-sim) which is used in this article is a model within the STAPM framework which simulates effects of tax and pricing policies on tobacco and/or alcohol consumption and computes consequent health and economic outcomes.

### Joint Tobacco and Alcohol Policy Modelling

TAX-sim models the dynamics of both alcohol and tobacco and integrates their disease epidemiology. This means that a longer disease list of 84 tobacco and/or alcohol related diseases are considered. While the present article is alcohol focused, the dynamics of the tobacco component of the model are still important as they drive mortality and morbidity outcomes.

### The STAPM Platform

The STAPM platform is a basis for three different models which are used in the appraisal of tobacco and alcohol tax and price interventions:

- [The Sheffield Alcohol Policy Model in R (SAPM-R)](https://stapm.gitlab.io/SAPM-R.html)
- [The Sheffield Tobacco Policy Model (STPM)](https://stapm.gitlab.io/STPM.html)
- [Tobacco and Alcohol Tax and Price Intervention Simulation Model (TAX-sim)](https://stapm.gitlab.io/TAX-sim.html)

The SAPM-R and STPM models respectively model the dynamics of alcohol and tobacco consumption, using an individual-level micro-simulation approach. The TAX-sim model considers both alcohol and tobacco consumption in the same model. It does this to enable fair comparisons between alcohol and tobacco policy, and it uses the underlying components of the SAPM-R and STPM models to inform the modelling of price and tax intervention effects. This supplementary appendix is produced from existing technical documentation which details the input data used and methodologies underpinning these three models. The key documents which this appendix draws on are:

- [TAX-sim technical report](https://osf.io/rwvdb)^1^
- SAPM-R technical report^2^
- STPM technical report^3^

## Model Overview

The STAPM method allows the simulation of entire populations at an individual level, tracking individual transitions in tobacco and alcohol consumption as they age. Individuals are indexed by age (single years, from 18 years to 89 years), sex and socio-economic conditions in terms of quintiles of the Index of Multiple Deprivation (IMDQ). The model is based on a synthetic population of representative individuals drawn from survey data. Simulated individuals are stratified into 800 population subgroups defined by age, sex, deprivation status measured by Index of Multiple Deprivation (IMD) quintile^4^, five categories of tobacco consumption (including non-smoker), and three drinker categories (moderate [no more than 14 units/week], hazardous [>14 units, but no more than 35 units for women and 50 units for men] and harmful [>35/50 units/week for women/men respectively]), plus abstainers. This detailed stratification allows population heterogeneity in purchasing preferences, prices paid, and risks of harm, which previous research suggests substantially affects the outcomes of alcohol pricing policies^5^.

### Description of the model structure

An overview of the model structure for the present analysis:

- Population: Scotland
- Age range: 18-89 years
- Index year (the year in which the model is initiated): 2017
- Policy effect year (the year in which the interventions are applied): 2019
- Time step: The simulation "clock" ticks forward one year at a time, i.e., *y* moves on to *y + 1*, and during that time the individual has his or her birthday and ticks onwards in age from *a* to *a + 1*.
- Time horizon (the year up to which the model is run): 2038
- Price year (the year that real terms prices correspond to): 2019

The 'index year' of the model is the year in which the model is initialised. The 'synthetic population' in the index year is the population sample of individuals aged 18-89 that provides the starting distributions of tobacco and alcohol consumption according to age, sex and Scottish index of multiple deprivation quintiles (IMDQ). A high-level schematic of the TAX-sim model used in this analysis is provided in Figure A1.


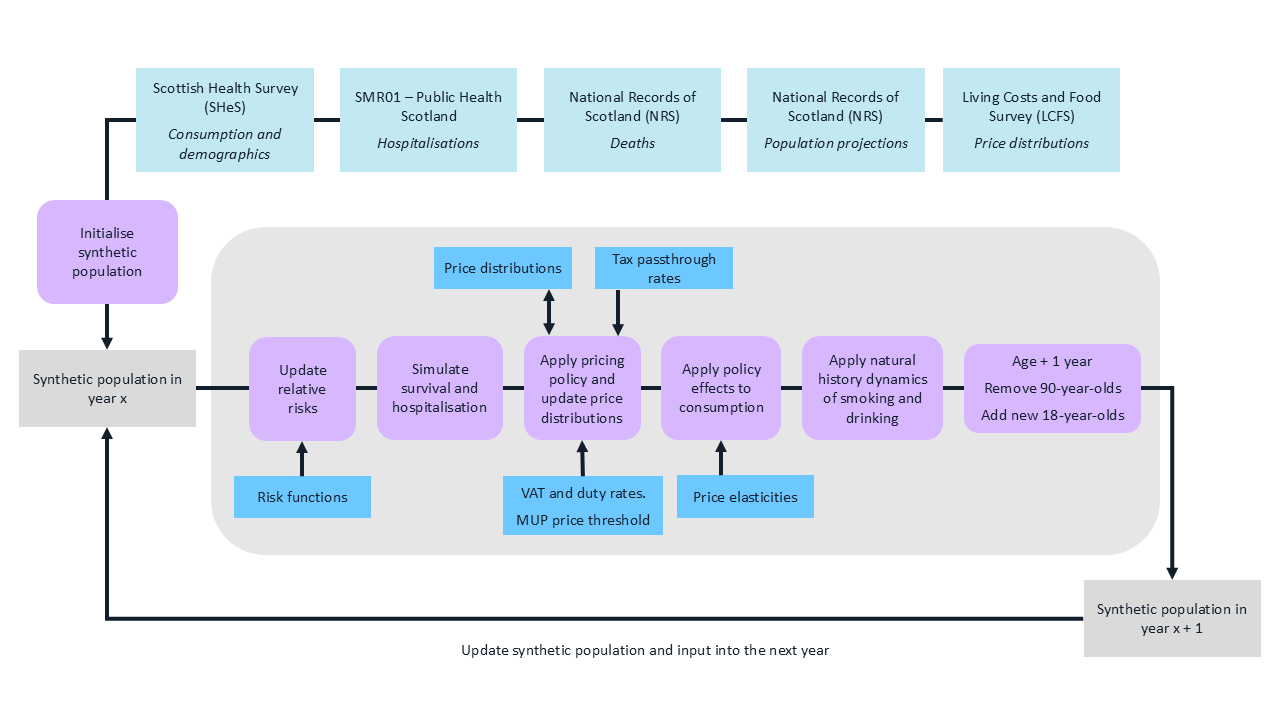


Figure A1 Schematic of the Tobacco and Alcohol Tax and Price Policy Simulation Model

The synthetic population in the index year is a weighted bootstrap sample with replacement of 200,000 individuals from the Scottish Health Survey (SHeS) years 2016 to 2018. The variables retained in the synthetic population are single year of age, sex, IMDQ, mean weekly consumption, and the percentage split between beers and ciders, wines, spirits, and ready-to-drinks (RTDs) i.e., alcopops. Information is also retained on tobacco consumption and smoking history to populate the tobacco component of the model. The next step is to bring in information from the Living Costs and Food Survey (LCFS) to split the SHeS combined beer and cider category into consumption of beer and cider separately, and to split the 5 alcohol beverage categories into 10 by differentiating between on-trade and off-trade consumption. Individuals are then put into the categories of level of smoking and levels of drinking. To minimise noise in the Monte Carlo simulation between the control and intervention arms, individuals are assigned a set of random seeds that minimise the random differences between the two arms.

### Initialising the model

#### Initial population

The "index year" of the model is the year in which the model is initialised. The index year could be set to correspond to the earliest year of available data, or to a policy-relevant year, e.g., the year before the policy change being modelled. The "synthetic population" in the index year is the population sample of individuals aged from the youngest age in the model (e.g. the legal drinking age in the UK of 18 years) up to a maximum age of 89 years. That sample of individuals provides the starting distributions of tobacco and alcohol consumption according to age, sex (m/f) and socio-economic conditions in terms of Index of Multiple Deprivation Quintiles (IMDQ). The number of individuals in the synthetic population in the index year can be controlled by a parameter set prior to model initialisation. The source population sample is then sampled in a bootstrap process with replacement until the specified number of individuals have been obtained. For example, a model might be initialised by sampling 200,000 individuals from a source sample of respondents to the Scottish Health Survey pooled across years 2016 to 2018.

The set of variables retained in the synthetic population are those that are needed to inform the simulation. The core variables for a model of alcohol consumption are age (in single years), sex, IMDQ, the average weekly consumption of alcohol in terms of UK standard units of alcohol per week (1 unit = 10ml or 8g pure ethanol), and the percentage split of individuals' alcohol consumption between beers, ciders, wines, spirits, and RTDs. The consumption of these 5 beverage types is further split by whether the alcohol consumption is consumed in the on- or off-trade channels.

The distribution of alcohol consumption across the resultant 10 beverage types is informed by using household survey information on alcohol consumption from national surveys for the three nations in Great Britain: The Health Survey for England (HSE), Scottish Health Survey (SHeS), and National Survey for Wales (NSW). These data are combined with information on individual purchasing patterns from the UK-wide Living Costs and Food Survey (LCFS). Specifically, the HSE does not separate beer from cider consumption and does not separate consumption between the on- and off-trades, and so this information is brought in from the LCFS.

As each year is simulated a new set of simulated individuals is "born" into the model aged $a_{min}$(18). These are then subject to the possibility of initiating smoking, and all the transitions and events described above as time runs forward on the simulation "clock".

#### Random number seeds

To minimise noise in the Monte Carlo simulation between the control and intervention arms, individuals are assigned a set of random seeds that minimise the random differences between the two arms. Everyone who enters the population simulation is assigned their own random number seed. Separate seeds are given for each process, e.g., a seed for simulating survival and a different seed for simulating transitions in alcohol consumption. The random seed for each individual is updated in each year of the simulation.

### Data sources

This section describes the use of data and parameters from published literature to inform the simulated population and outcomes.

#### Population size data

The data on mid-year population sizes for England and Wales was supplied by the Office for National Statistics, and for Scotland by National Records of Scotland. The data cover years from 2001 to the latest available for England and Wales, and from 2008 for Scotland. The counts are stratified by IMD quintiles, sex, age groups by single year of age then 90+ years.

Two forms of population count data are used: (1) the observed population stratified by age, sex and IMD quintiles; (2) the forecast population stratified by age and sex. For future years, population count data by age and sex are taken from the national primary population projections. An IMD quintile split is introduced into the forecast population counts by age and sex based on the IMD quintile split in the last year of observed data.

The population count data are used to calculate model inputs of the observed population rates of death, disease, and associated hospital admissions. The population count data are also used to inform the structure of the synthetic population with which the model is initialised. Population count data for the youngest age in the model (e.g. age 18) are used to inform the number of individuals who are added to the simulation in each year when a new birth cohort is added to the model. The number of new individuals added at the youngest age in each year is the number of individuals of that age expected to be in the population multiplied by a factor calculated for the year in which the model was initialised that scales between the actual and simulated population sizes.

#### Consumption data

Population survey data is used to inform the starting distribution of alcohol consumption among individuals who enter the microsimulation, and the subsequent dynamics of alcohol consumption considering trends over age, period, and birth cohort. For Scotland, data is used from the Scottish Health Survey (SHeS), a series of annual surveys covering health and health-related behaviours.

##### Drinking data

The average amount drunk in a week is estimated in terms of UK standard units of alcohol (1 unit = 10ml or 8g pure ethanol). Units are estimated separately for six beverage types within the SHeS: normal beer or cider, strong beer or cider, spirits, sherry, wine, and alcopops (also known as "ready to drink" or RTD). These are then grouped into four categories: beer (combining normal beer, strong beer), wine (combining wine and sherry), spirits, and alcopops. Data on the proportion of total alcohol purchasing accounted for by each beverage type in each population subgroup from the Living Costs and Food Survey (LCFS) is subsequently used to inform the splits of beer into beer and cider, and to split alcohol consumption between the on- and off-trade.

##### Smoking data

Cigarette smoking status is quantified in terms of self-reported data on whether an individual is a current, former, or never regular cigarette smoker, and if a former smoker how many years have passed since quitting. Smoking states are defined as follows:

- A never smoker is someone who has never smoked or has only tried a cigarette once or twice in their lifetime.
- A current smoker is someone who smokes cigarettes either regularly or occasionally.
- A former smoker is someone who used to smoke cigarettes either regularly or occasionally.

#### Living Costs and Food Survey

The Living Costs and Food Survey (LCFS) is a repeated cross-sectional survey of roughly 12,000 households randomly sampled from across the UK. The LCFS collects transaction-level information on spending patterns and the cost of living that reflect household budgets, using a 14-day diary of all spending by household members aged 16 or over. It is conducted throughout the year, across the whole of the UK. Transaction-level data from the LCFS is accessed securely through the UK Data Service Secure Lab. LCFS data are used to produce the price distributions with which the TAX-sim model is initialised. The procedure for deriving the price distributions is as follows:

Data preparation:

- The LCFS transaction-level data was cleaned and adjusted to 2016 prices. Each transaction was assigned a value and labelled if it fell into one of the 12 product categories of interest: beer, cider, wine, spirits, RTDs (for the on and off trade), factory made cigarettes, and roll your own tobacco.
- Each product was assigned into price per unit bins – 2p bins for tobacco and 10p bins for alcohol. For alcohol, a unit is 10ml/8g of ethanol or one UK unit of alcohol. For tobacco, a unit is one cigarette, or 0.5g of tobacco.
- With the data labelled in transaction level form, we next labelled each individual based on their socio-demographic characteristics.
  - Age: This is split into 4 categories: 18-24, 25-34, 35-49, 50+
  - Sex: Male and Female
  - Income quintile: Using the variable equivalised household income we created income quintiles labelled 1 to 5 where 1 was the poorest households and 5 was the richest in the entire sample.
- Following guidance by the UK Chief Medical Officer and previous work conducted within the SARG group at the University of Sheffield, we categorised individuals into 4 alcohol habit groups and 5 tobacco habit groups:
  - Alcohol drinker category (4 groups): Abstainer, Moderate drinker (purchased 0< to 14 units of alcohol), Hazardous (14-50 units for men, 14 -35 for women), and Harmful drinker (50+ for men and 35+ for women).
  - Smoker category (5 groups): Non-smoker, smokes 0 to 70 a week, 70 – 140 a week, 140 – 280 a week, and 280+.

Regression analysis:

- With each individual assigned their socio-economic status as well as their drinking and smoking characteristics, we used regression analysis to model the price distribution.
- We estimated the logit model individually for each of the 12 products to model the probability that a transaction occurred within a particular 10 pence price band for alcohol and 2 pence price band for tobacco. The regression model includes respondent's age-sex group, income quintile, drinker type (abstainer, moderate, hazardous, harmful) and smoker type (non-smoker, light, medium, and heavy smokers). The equation is therefore:

$$Prob\left( p_{it} \right)=f({Age}_{i},{Sex}_{i},{Income}_{i},{AlcStatus}_{i},{TobStatus}_{i})$$

- The model predicts the probability that the price paid is within a 10p or 2p band. Subscripts i, t, denote individual i making transaction t. It is modelled using an Ordered Logistic regression model which takes the ordered nature of the dependent variable into account when estimating the parameters attached to the independent variables. Age-Sex is a combination of 4 age groups and 2 sexes, and income is income quintile.
- Each model was estimated for 50 price bands.

Extraction and prediction:

- Once the model was run these coefficients were extracted from the UK Data Service Secure Lab, meeting their criteria for non-disclosive outputs. We then used the extracted coefficients to "predict" the shaped of the price distributions for each subgroup defined by age, sex, income quintile, drinker status, and smoker status. Again, this was estimated individually for the 12 products.

The final price distributions elicited from the analysis of LCFS for the off-trade alcohol products were calibrated to the MESAS monitoring report^6^ data on price distributions for 2017, to produce price distributions pre-MUP introduction in 2018 to initialise the model.

#### Mortality data

The model contains a total of 84 health conditions which are attributable to either alcohol, tobacco, or both and are listed in full in Table A1 (alcohol), Table A2 (tobacco), and Table A3 (both). “Acute” alcohol-related health conditions are related to high levels of alcohol consumption on single drinking occasions. For each of the tobacco and/or alcohol related health conditions, death counts, stratified by age, sex, and Index of Multiple Deprivation (IMD) quintile for Scotland were obtained from the National Records of Scotland (NRS). To address year-on-year variance in small counts, we smoothed the time trends across several years and used the smoothed figure as the model input. The mortality rate is quantified in terms of cause-specific central rates of death in one-year intervals of age and period, stratified by sex and IMD quintiles. These central death rates are converted to the probabilities of death in a one-year age interval to simulate individual deaths. They are also used to construct period lifetables from which the remaining expected years of life at each age are calculated to estimate the years of life lost to death in the STAPM simulation.

Table A1 Health conditions wholly or partially attributable to alcohol

| **Health Condition** | **Type** |
| --- | --- |
| Breast cancer | Cancers |
| Hypertensive diseases | Cardiovascular |
| Cardiac arrhythmias | Cardiovascular |
| Cirrhosis of the liver (excluding alcoholic liver disease) | Other |
| Acute pancreatitis | Other |
| Chronic pancreatitis | Other |
| Epilepsy and status epilepticus | Other |
| Transport injuries | Partially-attributable acute |
| Fall injuries | Partially-attributable acute |
| Exposure to mechanical forces (including machinery accidents) | Partially-attributable acute |
| Drowning | Partially-attributable acute |
| Fire injuries | Partially-attributable acute |
| Other unintentional injuries | Partially-attributable acute |
| Intentional self-harm | Partially-attributable acute |
| Assault | Partially-attributable acute |
| Other intentional injuries | Partially-attributable acute |
| Accidental poisoning by exposure to noxious substances | Partially-attributable acute |
| Alcoholic cardiomyopathy | Wholly attributable to alcohol |
| Alcoholic liver disease | Wholly attributable to alcohol |
| Acute pancreatitis (alcohol induced) | Wholly attributable to alcohol |
| Chronic pancreatitis (alcohol induced) | Wholly attributable to alcohol |
| Alcoholic gastritis | Wholly attributable to alcohol |
| Alcohol-induced pseudo-Cushing's syndrome | Wholly attributable to alcohol |
| Acute intoxication | Wholly attributable to alcohol |
| Mental and behavioural disorders due to use of alcohol | Wholly attributable to alcohol |
| Degeneration of the nervous system due to alcohol | Wholly attributable to alcohol |
| Alcoholic polyneuropathy | Wholly attributable to alcohol |
| Alcoholic myopathy | Wholly attributable to alcohol |
| Maternal care for suspected damage to foetus from alcohol | Wholly attributable to alcohol |
| Excessive blood level of alcohol | Wholly attributable to alcohol |
| Toxic effect of alcohol | Wholly attributable to alcohol |
| Alcohol poisoning | Wholly attributable to alcohol |
| Evidence of alcohol involvement determined by blood alcohol level | Wholly attributable to alcohol |

Table A2 Health conditions wholly or partially attributable to tobacco

| **Health Condition** | **Type** |
| --- | --- |
| Lung cancer | Cancers |
| Nasopharynx and sinonasal cancer | Cancers |
| Oesophageal AC cancer | Cancers |
| Stomach cancer | Cancers |
| Kidney cancer | Cancers |
| Lower urinary tract cancer | Cancers |
| Bladder cancer | Cancers |
| Cervical cancer | Cancers |
| Acute myeloid leukaemia | Cancers |
| Peripheral arterial disease | Cardiovascular |
| Abdominal aortic aneurysm | Cardiovascular |
| Venous thromboembolism | Cardiovascular |
| Ulcerative colitis | Other |
| Parkinson's disease | Other |
| Alzheimers disease | Mental health |
| Vascular dementia | Mental health |
| All-cause dementia | Mental health |
| Depression | Mental health |
| Schizophrenia | Mental health |
| Bulimia | Mental health |
| Psychosis | Mental health |
| Multiple sclerosis | Other |
| Systematic lupus erythematosis | Other |
| Low back pain | Other |
| Psoriasis | Other |
| Age-related macular degeneration | Other |
| Crohn's disease | Other |
| Hip fracture | Other |
| Rheumatoid arthritis | Other |
| Chronic kidney disease | Other |
| End-stage renal disease | Other |
| Senile cataract | Other |
| Hearing loss | Other |
| Chronic obstructive pulmonary disease | Respiratory |
| Asthma | Respiratory |
| Obstructive sleep apnoea | Respiratory |
| Idiopathic pulmonary fibrosis | Respiratory |

Table A3 Health conditions wholly or partially attributable to tobacco

| **Health Condition** | **Type** |
| --- | --- |
| Oral cavity cancer | Cancers |
| Pharyngeal cancer | Cancers |
| Laryngeal cancer | Cancers |
| Oesophageal SCC cancer | Cancers |
| Oesophageal cancer | Cancers |
| Pancreatic cancer | Cancers |
| Cancer of the liver and intrahepatic bile ducts | Cancers |
| Colorectal cancer | Cancers |
| Ischaemic heart disease | Cardiovascular |
| Haemorrhagic stroke | Cardiovascular |
| Ischaemic stroke | Cardiovascular |
| Type II diabetes | Other |
| Tuberculosis | Respiratory |
| Pneumonia | Respiratory |
| Influenza (clinically diagnosed) | Respiratory |
| Influenza (microbiologically confirmed) | Respiratory |

#### Hospital Episode Statistics

For each of 84 tobacco and/or alcohol related health conditions, hospital admission rates stratified by age, sex, and Index of Multiple Deprivation (IMD) quintile are calculated from individual patient records taken from the SMR01 admitted patient care data from Public Health Scotland.

Rates of hospital admission can be calculated using different methods ([see the discussion paper that we developed to understand the range of methods](https://stapm.gitlab.io/r-packages/hesr/articles/english_secondary_care_modelling_report.pdf)).

The version of the STAPM model used in this study uses a version of the “narrow” method to estimate rates of hospital admissions. The narrow rate is a more specific estimate of the rates of hospital admission for each condition per 100,000 people. The narrow rate looks only for tobacco and/or alcohol related diagnosis codes in the primary diagnostic position of an episode of care. Subsequent diagnostic positions are also scanned for external cause codes, e.g., assault, which do not feature in the primary diagnostic position but are important elements of the list of tobacco and/or alcohol related conditions considered in the model.

Three outcomes are produced from the hospital record data:

1. Morbidity rates per 100,000 (derived using NRS population estimates) for each health condition and age-sex-socioeconomic status subgroup. Year-on-year variance in small counts is addressed by smoothing the time trends in the calculated rates.
2. Unit costs of hospitalisations by condition, age group, sex and IMDQ. These costs are derived from the hospital episode-level HRG reference costs associated with the length of stay in hospital and the procedures applied. Currently our unit costs are calculated based on the 2016/17 data. Unit costs are inflated as necessary using the Hospital and Community Health Services (HCHS) pay and price inflation index.

## Microsimulation of alcohol consumption dynamics

This section describes the implications for the design of the alcohol model within STAPM of the need to align the method for simulating alcohol consumption with the method for simulating tobacco consumption. The starting point for the design of the alcohol model within STAPM was the technical methods used in the Sheffield team's existing alcohol model, SAPM version 4^7^.

SAPM4 models the effects of alcohol pricing policies on the distribution of prices for different types of alcohol and hence the average weekly amount of alcohol consumed. This model mechanism allows the computation of economic effects on consumer spending on alcohol, and industry and government revenue from alcohol sales. To model the effects on diseases related to acute alcohol intoxication, the effects of policies on average weekly alcohol consumption are subsequently linked to effects on the frequency and level of single occasion drinking^8,9^. To model the effects on chronic diseases, changes to the risk of chronic diseases due to changes to average weekly alcohol consumption are calculated considering the lagged effects of changes to patterns of alcohol consumption over many years^10^.

SAPM4 simulates the effects of alcohol price changes on alcohol consumption in a repeated cross-sectional sample of individuals over the next 40 years. It does not simulate the effects of price changes on the individual-level dynamics of alcohol consumption as they age. This means that, in the absence of a tax change, the size, composition, and alcohol consumption of the modelled population remains unchanged over the next 40 years. The main change required to align the method for alcohol with the method for tobacco in STAPM was to move the simulation approach from repeat cross-sections to individual-level dynamics with age. The main reason for this change was that simulating the individual-level dynamics of tobacco initiation, quitting and relapse is so important for the accurate prediction of policy effects that it was not plausible to assume that alcohol consumption did not also follow an individual-level dynamic.

### Alcohol consumption

Alcohol consumption data is prepared for the model based on the method used to inform the original Sheffield Alcohol Policy Model. A standard set of code is now used for processing these data in a consistent way for England, Scotland, and Wales that is stored and version controlled within the hseclean R package^11^. The code in that R package is open source, each function within the code is documented, there are vignettes that explain the methods for data processing, and there are "data check" reports that we produce to help quality assure the processing of the data (e.g. [the data check for Scotland alcohol data](https://stapm.github.io/hseclean/articles/Scotland_alcohol_data_report.html)).

The average amount drunk in a week is estimated in terms of UK standard units of alcohol (1 unit = 10ml or 8g pure ethanol) and is based on survey questions measuring participants frequency and quantity of consumption.

*Whether someone drinks and frequency of alcohol consumption*: Adults are classed as drinkers if they reported consuming alcohol at all in the last 12 months, even if reporting only having 1-2 drinks a year. Drinking frequency is converted to a numerical variable as follows:

- "Almost every day" = 7 days a week
- "Five or six days a week" = 5.5 days a week
- "Three or four days a week" = 3.5 days a week
- "Once or twice a week" = 1.5 days a week
- "Once or twice a month" = 0.375 days a week
- "Once every couple of months" = 0.188 days a week
- "Once or twice a year" = 0.029 days a week

*Quantity of alcohol consumption:* Participants report their alcohol consumption on a typical drinking day across six beverage categories: normal beer, strong beer, spirits, sherry, wine and RTDs. The questionnaire also allows participants to report their consumption in a range of container types and sizes and, we assume the following sizes for these containers: normal and strong beer or cider (half pint 284ml, small can 330ml, large can 440ml, bottle 330ml), spirits (serving 25ml), sherry (serving 50ml), wine (small glass 125ml, standard glass 175ml, large glass 250ml, bottle 750ml), and alcopops (small can 250ml, small bottle 275ml, large bottle 700ml). Alcoholic strength is based on the following assumed alcohol-by-volumes (ABVs): normal beer or cider (4.4%), strong beer or cider (8.4%), spirits (38%), sherry (17%), wine (12.5%) and RTDs (4.5%). For each beverage type, we then calculate the number of weekly units consumed for each beverage type by multiplying the volume of product consumed on a typical day by the ABV (e.g. a 12% ABV 750ml bottle of wine contains 0.125 * 750 = 90ml of alcohol, or 9 units) and then multiplying this by the frequency of consumption.

The data in SHeS does not distinguish between consumption in the on-trade (consuming on-premises in pubs, bars, restaurants) and the off-trade (consumption at home of alcohol purchased in supermarkets, shops, and off-licences). It also does not distinguish between beer and cider, combining both beverages into a single category. The more detailed consumption data in the LCFS is used to estimate consumption in the SHeS separately for beer, cider, wine, spirits, and RTDs in both the on-trade and off-trade. To separate beer and cider, the LCFS data from 2014-2017 is used to calculate the proportionate split of beer and cider from the combined total consumption of both beverages. The LCFS is then used in a similar manner to calculate, for each of the five beverages, the proportionate split between on and off-trade consumption. These calculations are stratified by income quintile, sex, and drinker category. The result is a vector of ten percentages which sum to 100% describing the distribution of total alcohol consumption across the 10 beverage categories in the model for the 30 population subgroups defined by income, drinker category, and sex.

The calculation of total weekly alcohol consumption therefore has the following steps:

1. Convert the categorical variables to numeric variables for the frequency with which each beverage is typically consumed (normal beer or cider, strong beer or cider, spirits, sherry, wine, alcopops).
2. Convert the reported volumes usually consumed (e.g., small glass, large glass) into volumes in ml, using the beverage size assumptions above. In doing so, variations in recording among years and between the interview and self-complete questionnaire are accounted for.
3. Combine the volumes (ml) usually consumed with the frequency of consumption to give the average volume of each beverage type drunk each week (assuming constant consumption across the year).
4. Convert the average volumes of each beverage consumed each week to UK standard units of alcohol consumed, using the alcohol content assumptions above.
5. Collapse normal and strong beer or cider into a single "beer or cider" variable by summing their units. Collapse wine and sherry into a single "wine" variable by summing their units.
6. Calculate total weekly units by summing across beverage categories.
7. Cap the total units consumed in a week at 300 units as estimates of variation in consumption are less reliable.

Individuals in the simulation are categorised into drinker groups based on the amount of total weekly consumption of all alcoholic beverages. The four categories are as follows:

- Abstainer - 0 units / week.
- Lower-risk / moderate drinker - less than 14 units / week.
- Increasing-risk / hazardous drinker - 14 or more units / week and less than 35 units (females) or less than 50 units (males).
- Higher-risk / harmful drinker - 35 or more units / week (females) or 50 or more units / week (males).

Note that a key difference between SAPM and STAPM is that in SAPM individuals remain categorised by their baseline levels of alcohol consumption, whereas in STAPM an individuals' drinker category is dynamically updated in the simulation according to current consumption. See Section 1.3.5 for the implications of this for interpreting model outcomes.

#### Upshifting to account for under-reporting of consumption

It is generally accepted that self-reported data on alcohol consumption underestimates true consumption by as much as 50%^12^. While the base case for the SAPM-R simulation is to use the self-reported consumption from the survey data, the model can apply an "upshift" factor to self-reported consumption to account for this underestimation so that it covers 80% of total alcohol sales, in line with the Global Burden of Disease approach. [See here](https://stapm.github.io/hseclean/articles/Scotland_alcohol_upshifting_report.html) for an illustration of the impact of applying this upshifting to the distribution of alcohol consumption for Scotland.

### Participation in alcohol consumption

The dynamics of alcohol consumption in STAPM are modelled as two components: (i) participation in alcohol consumption - the proportion of people in the current drinker vs. abstainer groups and (ii) conditional consumption - the average number of units consumed per week by people who drink. The dynamics of alcohol consumption are modelled from the minimum age set in the model (e.g. 18 years) to 89 years.

In the microsimulation, alcohol participation is adjusted to correspond to the estimated population-level trends in the proportion of people who drink by age and calendar year for each sex and IMDQ group. The method to do so was based on an existing SAPM method to change levels of participation and conditional consumption to represent estimated trends by age, period, and birth cohort (APC) up to 2035^13,14^.

The method used has three parts:

1. Estimate the reference proportions of people who drink for input to the model - Proportions of people who drink are estimated from the health survey data for each calendar year, single year of age, sex and IMDQ. The variation in these proportions by calendar year and age is smoothed using a moving average computed by sampling from a 3x7 moving window of calendar year and age. This method assumes that the change in proportions from age a and year y to age a+1 and year y+1 accurately represents the net of individual behavioural transitions between the drinker and abstainer states. However, this is unlikely to be the case without adjustments for bias, e.g. the influences of misreporting of alcohol consumption and demographic change including the effects of selective mortality by drinking state. The method developed to estimate smoking state transition probabilities for the STAPM model does include adjustments for bias. Future versions of STAPM will explore improvements to the method of informing the simulated change to alcohol participation.
2. Adjust participation in the model - The simulation cycles through each age for each sex and IMDQ. The proportion of people in the simulated population who are drinkers is calculated for age a and year y. The proportion of people in the simulated population who should be drinkers at age a+1 and y+1 is determined by:

(i) Looking up the reference proportions of drinkers for age a and year y, and age a+1 and year y+1.

(ii) Calculating the ratio between these reference proportions, which gives an estimate of the expected relative change in the proportion of people who are drinkers between ages a and a+1 in years y and y+1.

(iii) Adjust the calculated proportion of people who are drinkers from the simulated population by multiplying it by the relative change calculated in (ii).

For this study, there is no change in the reference proportions by calendar year, so the only variation in alcohol participation that influences the projected trend is that between ages for the index year.

1. Simulate turning peoples' alcohol consumption on or off - The number of people whose alcohol consumption needs to be turned on or off in the simulation is determined by multiplying the difference between the current and adjusted proportions of drinkers by the number of people in the simulation population for each year, age, sex and IMDQ. If the number of drinkers is falling, as indicated by a negative sign to the number of individuals who need to change, then the individuals to become non-drinkers are randomly sampled and their average weekly alcohol consumption set to zero. If the number of drinkers is rising, then the individuals to become drinkers are randomly sampled. These new consumers are assigned a value for units of alcohol consumed that is sampled from the distribution of units consumed for age a+1 in year y of the simulation for the corresponding sex and IMDQ. This means that if there has been a policy induced change to the distribution of alcohol consumption, then the alcohol consumption assigned to new drinkers will reflect that change.

### Alcohol conditional consumption

The method to track the life-course dynamics of the average number of units drunk per week by individuals who remain as current drinkers between years of the microsimulation is outlined below.

Individuals entering the simulation as drinkers are assigned an amount drunk based on the distribution of amount drunk in the current year, according to their age, sex and IMDQ. Individuals who remain as drinkers between successive years of the simulation have the amount that they drink updated to represent the expected change over age. The assumption is that each drinker remains at approximately the same percentile of the distribution of amount drunk as they age. The shape of the distributions of the amount drunk is estimated from the same health survey sample used to inform the initial synthetic population. Distributions are estimated as empirical cumulative distribution functions (ecdf) corresponding to each calendar year, single year of age, sex and IMDQ. Estimates of the distribution of amount drunk are 3x7 year rolling average distributions by year and age. For this study, there is no change in the reference distributions by calendar year, so the only variation in alcohol conditional consumption that influences the projected trend is that between ages for the index year.

The update of amount drunk with age cycles through each single year of age for each sex and IMDQ subgroup, as follows:

1. Define the donor values and percentiles – Define a 'mesh' of possible values of consumption that an individual could transition to at age a+1. For example, the mesh for the average number of units consumed per week could be a sequence of length 2,500 that ran from 0.01 to 130 units/week, which would give a mesh width of approximately 0.05 units per week. Using the ecdf of consumption corresponding to age a+1, each value in the mesh is assigned a percentile in the distribution of consumption. This produces a 'donor' set of percentiles corresponding to the possible new values of consumption.
2. Define the reference values and percentiles - Look at the average amount drunk in the simulated population at age a. Using the ecdf of consumption corresponding to age a, drinkers are assigned a percentile in the distribution of consumption. This produces a 'reference' set of percentiles for the drinkers at age a.
3. Match the donor and reference percentiles and update individual consumption - For drinkers aged a, determine the nearest neighbour match between their reference percentile and the donor percentiles. Assign individuals the new value of the average amount drunk per week at age a+1 that corresponds to the selected donor percentile.

### Alcohol product preferences

Changes to a 10-value preference vector that gives the proportional split of the average number of units drunk per week between beer, cider, wine, spirits and RTDs in the on- and off-trade are simulated as drinkers age. The new preferences for age a+1 are sampled from the distribution of preferences of drinkers in the current year of the simulation within each drinker group, sex and IMDQ subgroup. This means that the subgroup distributions of product preferences are preserved between years of the simulation but that each drinker moves at random within the distribution of preferences for their drinker group, sex and IMDQ subgroup as they age.

### Alcohol consumption outcomes

When reporting the changes to alcohol consumption that result from a policy change or intervention, it is important to understand a key difference between the STAPM model and the original SAPM model used in previous reports. In SAPM, individuals were assigned to drinker groups based on their alcohol consumption at baseline and remained in these groups throughout the modelling process. We then reported the average change in consumption and other outcomes within each group, which reflected changes in consumption experienced for individual drinkers who began in each group at baseline. The structure of SAPM-R allows a different approach where we track changes in individual drinkers' alcohol consumption over time and allow drinkers to move between drinker groups in line with these changes. This means that a drinker consuming 16 units per week at baseline will be classified as a hazardous drinker, but if the introduction of a policy leads them to reduce their drinking to 12 units per week, they will be reclassified as a moderate drinker from that point onwards. It is therefore possible that the number of harmful drinkers in each arm will be different as more individuals may have transitioned between drinker groups in one scenario than the other. As a result, the difference in mean consumption for each drinker group between the intervention and control arms reflects changes in consumption among drinkers who remain in the group and the effect of drinkers moving into and out of the group.

For example, consider the following example that resulted from a policy analysis using the STAPM methods. At the population level, the intervention reduced alcohol consumption by 0.8 units per week, a relative reduction of 6.7%. However, the modelled consumption reductions within each of the three drinker groups were markedly smaller than this (-0.3% for moderate drinkers, -1.1% for hazardous drinkers and -2.6% for harmful drinkers). This is because the changes in consumption resulting from the intervention led some harmful drinkers to reduce their consumption sufficiently that they were reclassified as hazardous drinkers, and some hazardous drinkers to similarly reduce their drinking to the point where they became moderate drinkers. The modelled effects of the intervention were that 26,753 drinkers moved from the harmful to hazardous groups and 68,508 moved from hazardous to moderate. These figures represent a reduction of -18.6% in the number of harmful drinkers, a -4.8% reduction in the number of hazardous drinkers and a corresponding 2.7% increase in the number of moderate drinkers.

As the people moving into lower consuming groups are likely to be among the least heaviest drinkers within those groups (i.e. the people more likely to move to a lower drinking category will be people who drink at levels closer to the category boundary), this means the average in each group is pushed upwards (i.e. when relatively low consuming individuals leave a drinking category, the average amount drunk in that category increases). So even though the consumption of all individuals, and the overall population has fallen, this effect drags the mean consumption in both hazardous and moderate groups up. This drag offsets the fall in consumption among the individuals who remain within the group and is what leads to the lower within-group changes that we saw in the above example compared to the overall population. This effect is a version of what is sometimes referred to as 'Simpson's paradox', whereby splitting a population into subgroups leads to subgroup-level patterns that do not reflect the overall population pattern.

The difference in effect of an intervention on mean alcohol consumption at drinker group level between the original SAPM and the new SAPM-R (i.e. mean consumption within drinker groups going up in SAPM-R rather than down in SAPM) represent the fundamentally different way in which these models work. Effects on alcohol consumption from the SAPM-R model could therefore be misinterpreted if compared to similar numbers produced from the original SAPM model. The key to accurately interpreting the effects on alcohol consumption from SAPM-R is to consider the changes to mean alcohol consumption within a drinker group alongside changes in the number of drinkers in each group.

Despite these complexities, the results in the new SAPM-R structure have the advantage of allowing the model to report changes in the number of drinkers in each group arising from an intervention, something we have not previously been able to report.

## Modelling the relationship between consumption and harms

An epidemiological approach is used to model the relationship of changes in tobacco and alcohol consumption to changes in mortality and morbidity outcomes. The changes in tobacco and alcohol consumption that are defined within the model to affect mortality and morbidity are:

- Changes in smoking state, with lagged effects of quitting smoking.
- Changes in average weekly amount drunk, with lagged effects of changes in consumption.

### Relative risks of disease

STAPM considers the effects of tobacco and alcohol consumption on the risks of developing the ICD-10 defined categories of disease in Table A1, Table A2, and Table A3. In each year of the simulation, individuals in the simulated population are assigned a relative risk of disease based on their smoking and drinking behaviour and history, with risks differentiated by age and sex where this information in available.

The alcohol modelling considers 45 categories of adult diseases related to alcohol consumption and the corresponding dose-response effects of current levels of alcohol consumption on the relative risks of disease. The model links average weekly alcohol consumption to the risk of chronic diseases, and the amount of alcohol consumed on single drinking occasions to health harms associated with intoxication. Individuals' relative risk for each disease is calculated for four categories of disease:

- Chronic diseases partially attributable to long-term average levels of alcohol consumption – see Angus et al. for the list of diseases, sources of risk functions and their corresponding curves^17^. For some conditions there are separate risk functions for mortality and morbidity. For conditions that show a J-shaped risk function this indicates protective effects of alcohol for levels of alcohol consumption where the relative risk is less than 1.
- Chronic diseases wholly attributable to long-term average levels of alcohol consumption.
- Diseases partially attributable to acute alcohol consumption.
- Diseases wholly attributable to acute alcohol consumption.

The smoking modelling considers 52 categories of diseases related to smoking in adult smokers and the corresponding relative risks of these diseases in current vs. never smokers, and in former smokers according to the time since they quit^18^. There are 15 diseases in our combined list of 84 diseases that are related to both alcohol and tobacco (Table A3).

For oral cavity, pharynx, larynx and oesophageal cancers there is information from meta-analyses of interactions between tobacco and alcohol disease risk. This evidence shows that the risk of disease in someone who consumes both tobacco and alcohol is higher than would be expected from combining the independent risks from tobacco and alcohol. This additional risk due to tobacco-alcohol interaction is expressed as a "synergy factor"^19,20^. The effects of including the synergy factors are not included in the base-case results of the STAPM modelling – in the base-case the relative risks for drinking and smoking are combined as follows:

$${RR}_{combined}=1+(\left( {RR}_{alcohol}-1 \right)+\left( {RR}_{tobacco}-1 \right))$$

#### Chronic diseases wholly attributable to long-term average levels of alcohol consumption

The diseases in this category are: Alcoholic cardiomyopathy, Alcoholic gastritis, Alcoholic liver disease, Acute pancreatitis alcohol induced, Chronic pancreatitis alcohol induced, Alcohol induced pseudo-Cushing's syndrome, Alcoholic myopathy, Alcoholic polyneuropathy, Maternal care for suspected damage to foetus from alcohol, Degeneration of the nervous system due to alcohol, Mental and behavioural disorders due to use of alcohol^17^

STAPM uses the SAPM method to determine the shape of the risk function (the method is described on p28 in the Purshouse et al.^21^ modelling report for NICE). Briefly, for wholly attributable chronic conditions, risk is defined as the difference between mean daily consumption and a lower threshold below which risk is assumed to be equivalent to that of abstainers. The thresholds used are 2 units/day for both females and males (equivalent to 14 units/week). Below these thresholds risk is assumed to be 0.

#### Diseases partially attributable to acute alcohol consumption

Diseases in this category are injuries, e.g., traffic accidents, assault and falls, that are linked to the amount consumed on single drinking occasions and the subsequent amount of time that individuals remain intoxicated with a percentage blood alcohol content (%BAC) greater than zero. The STAPM method to calculate the relative risk of injuries for each drinker builds on the method used in SAPM4 - detailed description of the method can be found elsewhere^8,9^ and the method is summarised below.

Calculate the patterns of single occasion drinking for individuals in the simulation - In each one-year time step of the STAPM simulation, individuals' average weekly alcohol consumption is converted to estimates of:

- The frequency of drinking occasions (defined as n, or number of drinking occasions per week)
- The distribution of the amount of alcohol consumed on each occasion – this is a vector of probabilities that each integer number of grams of ethanol per day from 1-600g/day is consumed on a single drinking occasion. For each individual, alcohol consumption on a given drinking occasion is assumed to follow a normal distribution with mean of µ and standard deviation of $\sigma$. It is computed from the:
  - mean level of alcohol consumption for a given drinking occasion (defined as µ, or units of alcohol)
  - the variability of alcohol consumption for a given drinking occasion (defined as $\sigma$, or standard deviation of units of alcohol consumed in drinking occasions).

The parameters used in the calculation of patterns of single occasion drinking come from Hill-McManus et al.^8^ who analysed drinking occasions using data from detailed diaries in the National Diet and Nutrition Survey 2000/2001. The parameters allow prediction of an individual's characteristics of single occasion drinking from their average weekly alcohol consumption and a range of other characteristics that include age, income, employment, ethnicity, age left education, and social class. It was necessary to get the parameters into a form in which they fit with the age, sex and IMDQ stratification of the STAPM model. To do so, an additional computation was conducted to prepare the parameter inputs for the STAPM model. Specifically, the parameters from Hill-McManus were assigned to individuals in the HSE 2011-2017 (England), and SHeS 2008-2019 (Scotland) based on the full range of individual characteristics included in the statistical models. Weighted averages of these parameters were then calculated to give STAPM model inputs for each age category, sex and IMDQ.

Estimate annualised relative risk of alcohol-attributable injuries for individuals in the simulation - The duration of intoxication corresponding to the amount of alcohol drunk on an occasion is defined in terms of the time, in hours, after a drinking occasion that it would take for an individual's %BAC to drop to zero. The calculation of this time considers individuals' sex, height, and weight^22,23^. The rate at which the liver clears alcohol from the body is assumed to be 0.017 %BAC per hour. The probability that each level of alcohol is consumed on a drinking occasion is multiplied by the expected duration of intoxication for each amount of alcohol drunk on an occasion. The result of that calculation is then multiplied by the expected number of drinking occasions per week and by 52 weeks in a year. The annualised relative risk is calculated by summing the relative risk of injury across the year - the time during the year that is spent intoxicated is associated with the relative risks for the amount drunk on the occasion from Cherpitel et al. ^24^ , and the remaining time in the year that is not spent intoxicated has a relative risk of 1.

#### Diseases wholly attributable to acute alcohol consumption

Diseases in this category are those that can only be caused by acute alcohol consumption, e.g., alcohol poisoning. The STAPM method to calculate how the risk of these acute harms varies with the level of consumption on a single drinking occasion builds on the SAPM method. Due to the harms being wholly attributable to alcohol, no cases are expected in people who consume below a certain threshold of alcohol on a single occasion, i.e., there are no cases in the non-exposed reference group. The starting point for the computation is the probability distribution over the number of units of alcohol that could be consumed on a single drinking occasion. Values in the probability distribution corresponding to numbers of units below the typical threshold used for classifying heavy episodic drinking in the UK (3 units a day for women, 4 units a day for men) are set to zero. The probability distribution is then used to compute the total number of units above the thresholds expected to be drunk in a year. Risk is assumed to be proportional to that value.

#### Lag times from changes in alcohol consumption to changes in chronic disease risk

When modelling the link between alcohol consumption and the risk of chronic disease over many years, an important input is the assumption surrounding the 'time lag' – the time needed to achieve the full effect of a change in consumption on a change in disease risk. SAPM4 uses estimates of the temporal relationship between changes in consumption and the proportional reduction in the relative risk of disease, assuming that the full impact of a change in consumption on risk occurs by 20 years after the change in consumption (Holmes et al. 2012^10^) . The SAPM4 method needed to be adapted for STAPM to suit the STAPM model structure of simulating individual life-course trajectories of alcohol consumption. This meant that adding memory to track individual histories of relative risk for each disease over their life-courses.

STAPM stores individual risk histories for up to 20 years. In each year of the simulation, the stored risk histories are filtered to retain only the individuals currently present in the simulation. The relative risk of each disease assigned to an individual in the current year of the simulation is adjusted to take account of the stored risk history. For each annual stored risk value, the time difference to the current year of the simulation is calculated and the corresponding proportional reduction in the relative risk of disease is merged into the data. The adjusted relative risk of disease for the current year of the simulation is computed as a weighted average of the relative risks for past years for which the individual was tracked, where the weights correspond to the proportional reduction in relative risk. This means that the relative risk for the current year always has the lowest weight, which reflects the gradual emergence of the effects of changes in consumption on the risk of disease.

### Calculating population attributable fractions to estimate the total avoidable burden of mortality and morbidity due to tobacco and alcohol in a single year

This section describes the method to estimate Population Attributable Fractions (PAFs) - the proportions of cases of tobacco and/or alcohol related diseases that could be prevented if exposure to tobacco and alcohol in the population were removed^27,28^.

PAFs are estimated to show the potential impact of removing exposure to tobacco only, alcohol only and both tobacco and alcohol. The joint tobacco and alcohol PAFs tend to be smaller than the sum of the tobacco only PAF and the alcohol only PAF – because some cases of disease would require the removal of exposure to both tobacco and alcohol to be prevented, i.e., single substance PAFs over-estimate by not considering the remaining influence of other exposures. The estimated effects of removing only tobacco or only alcohol are therefore adjusted downwards so that they sum to the estimated effect of removing both tobacco and alcohol.

Formula for the PAF – the formula used in this project has two inputs: (1) data on the distribution of tobacco and alcohol consumption among individuals in a cross-sectional sample; (2) estimates of how the relative risk of disease varies with tobacco and alcohol consumption. It is defined for each disease as:

$$PAF= \frac{\sum_{i} P_{i}({RR}_{i}-1)}{1+\sum_{i} P_{i}({RR}_{i}-1)}$$

where **i** is an index of exposure to tobacco and/or alcohol, and RR is the relative risk of disease. PAFs might be estimated using the PAF equation separately for calendar years, ages or various definitions of population strata. For some diseases, the relative risks indicate that some levels of exposure to tobacco or alcohol consumption protect against the disease, i.e., that some smokers or drinkers are less likely to get the disease. Protective effects are defined by RR < 1, which when the PAF equation is applied results in negative PAFs.

### Linking behaviour change to change in mortality and morbidity between years in the microsimulation

This section describes how in STAPM changes in tobacco and alcohol consumption are linked to changes in mortality and morbidity rates each time the simulation "clock" ticks forwards one year. Annual updates to mortality and morbidity occur in both the control and intervention arms of the model, which means that when a tax change is applied in the intervention arm, its effects on tobacco and alcohol consumption cause mortality and morbidity divergence between model arms. The rates of mortality and morbidity that are updated are disease-specific and stratified by population subgroups defined by combinations of single years of age, sex and IMDQ.

The method applied in STAPM to link changes to consumption to changes to the rates of mortality and morbidity builds on the method applied previously in the SAPM model (see Section 2.3 in Brennan et al.^7^ which built on previous methods e.g. Gunning-Schepers^29^. All methods use the same basic approach, which is based on the notion of the population-attributable fraction. The rates of mortality and morbidity are updated in each year of the model simulation according to a proportional factor of change in the average relative risk of disease within each population subgroup. The proportional factor of change is the ratio of the average relative risks in the subgroup to be updated in the current year of the simulation relative to the previous year of the simulation. This proportional factor of change is known as the 'potential impact fraction' (PIF) or 'trend impact fraction^29^.

The PIF is calculated for each disease for mortality or morbidity using the formula:

$$PIF\left( y,j \right)=1- \frac{{RR}_{average}(y,j)}{{RR}_{average}(y-1,j)}$$

where y indicates the current year of the simulation and j indicates each age, sex, and IMDQ subgroup. The rates of mortality and morbidity from each tobacco and/or alcohol-related disease are updated in each year of the model simulation according to:

$$m\left( y,j \right)=m\left( y-1,j \right)[1+PIF\left( y,j \right)]$$

where **m()** is a generic notation for either the rates of mortality or morbidity.

### Mortality analysis

The starting point for the analysis of mortality outcomes is disease-specific mortality, defined as central rates of death in one year intervals of age and calendar year, stratified by sex and IMDQ

Calculate individual probabilities of death – In each year of the simulation, a probability of death for each individual is calculated. This probability of death incorporates mortality from tobacco and/or alcohol related diseases and from all other diseases not related to tobacco or alcohol consumption. The calculation of individual probabilities of death is based on a method used in the SAPM model, which can be summarised briefly as follows:

- Assigning individuals a relative risk for each tobacco and/or alcohol related disease based on their consumption status.
- Standardise the distribution of individual relative risks of disease so that they sum to 1 for each combination of age, sex and IMDQ subgroup.
- Multiply the disease-specific mortality rate for each age, sex and IMDQ subgroup by the standardised individual relative risks of disease and divide this by the subgroup average of the standardised individual relative risks.
- The result is individualised estimates of disease-specific mortality rates, which average to equal the subgroup mortality rate.
- The calculation assumes that the only cause of mortality variation among individuals within a year, age, sex and IMDQ subgroup is their tobacco and/or alcohol consumption.
- Compute the average cause-specific mortality rate according to levels of tobacco and alcohol consumption, age, sex and IMDQ – for use in reporting of model outputs.
- Convert the individualised estimates of cause-specific mortality to cause-specific probabilities of death for each individual during the year, assuming that mortality is constant during each one-year age interval.
- Sum the probabilities of death across causes – for subsequent use in simulating which individuals die in each year of the simulation.

Simulate individual deaths - The probability of death calculated for each individual is used to Monte Carlo simulate who dies by drawing from a binomial distribution, and the individuals who die are removed from the simulated population. The number of individuals who die in each year of the simulation for each age, sex and IMDQ subgroup is recorded. Those individuals still alive after the simulation of deaths then form the population for the next year of the simulation and the sequence repeats.

Calculate effects of the intervention on deaths and years of life lost to death – After the model simulation has run, 'post-processing' is conducted to compute the effects of the intervention on lifetable outcomes for:

- Number of years of life lived ("life-years") - shown by effects on the simulated population size, since the simulation moves forward in one year time intervals.
- Number of deaths in each year - estimated either from the number of deaths recorded in the model simulation, or from the estimated mortality rate and population size (the latter will be subject to less stochastic variation).
- Number of years of life lost due to deaths from tobacco- or alcohol-related causes - estimated by multiplying the number of deaths recorded at each age, in each sex and IMD quintile stratum by the expected remaining years of life for someone of that age in that stratum. The remaining expected years of life are therefore influenced by the modelled age- and stratum-specific mortality rates, i.e., both number of deaths and expected remaining years of life can respond to a policy change.

The effect of the intervention is then calculated as the annual or cumulative annual difference between these outcomes for the control and intervention arms.

### Morbidity analysis

All morbidity rates and outcomes are estimated using data from the Admitted Patient Care portion of the Scottish SMR01 data (section ‎1.2.3.5). After the model simulation has run, 'post-processing' is conducted to compute the effects of the intervention on outcomes for numbers of hospital admissions, and NHS costs of hospital admissions.

The methods to do so build on the approach taken by previous SAPM modelling with adaptations to ensure that policy effects on the above outcomes were comparable for tobacco and alcohol. These adaptations mean that the rates and unit costs used in the STAPM modelling differ slightly from those in the SAPM modelling. Where method changes were made, a conservative approach was taken such that STAPM tends to generate lower cost estimates than SAPM. For the rates of hospital admission, this more conservative approach corresponds to using a more narrowly defined method for assigning a hospital admission to a tobacco or alcohol related condition.

NHS costs of hospital admissions - Each hospital admission associated with a health condition is then assigned an average cost for the admission, where the costs of admissions are calculated considering only episodes of care that share a primary diagnosis with the diagnosis assigned to the admission (again, this is in keeping with our conservative approach to costing). New methods were added for STAPM that improve our estimation of the change in the NHS costs of hospital admissions by linking the Healthcare Resource Group fields to standard NHS intervention costs. Costs are inflated to 2022 prices using the Hospital and Community Health Services (HCHS) index. Costs are stratified by condition, sex and IMDQ.

## Modelling the effects of pricing policy on prices and consumption

This section describes the use of the TAX-sim model to simulate the impact of policies which aim to change the price of alcohol. TAX-sim models the impact of these policies on prices and subsequent effects on consumption. The effects on consumption impact on health outcomes through the mechanisms previously described. Figure A2 presents a schematic of this component of the modelling. The content of this section draws on the separate TAX-sim technical report^1^.


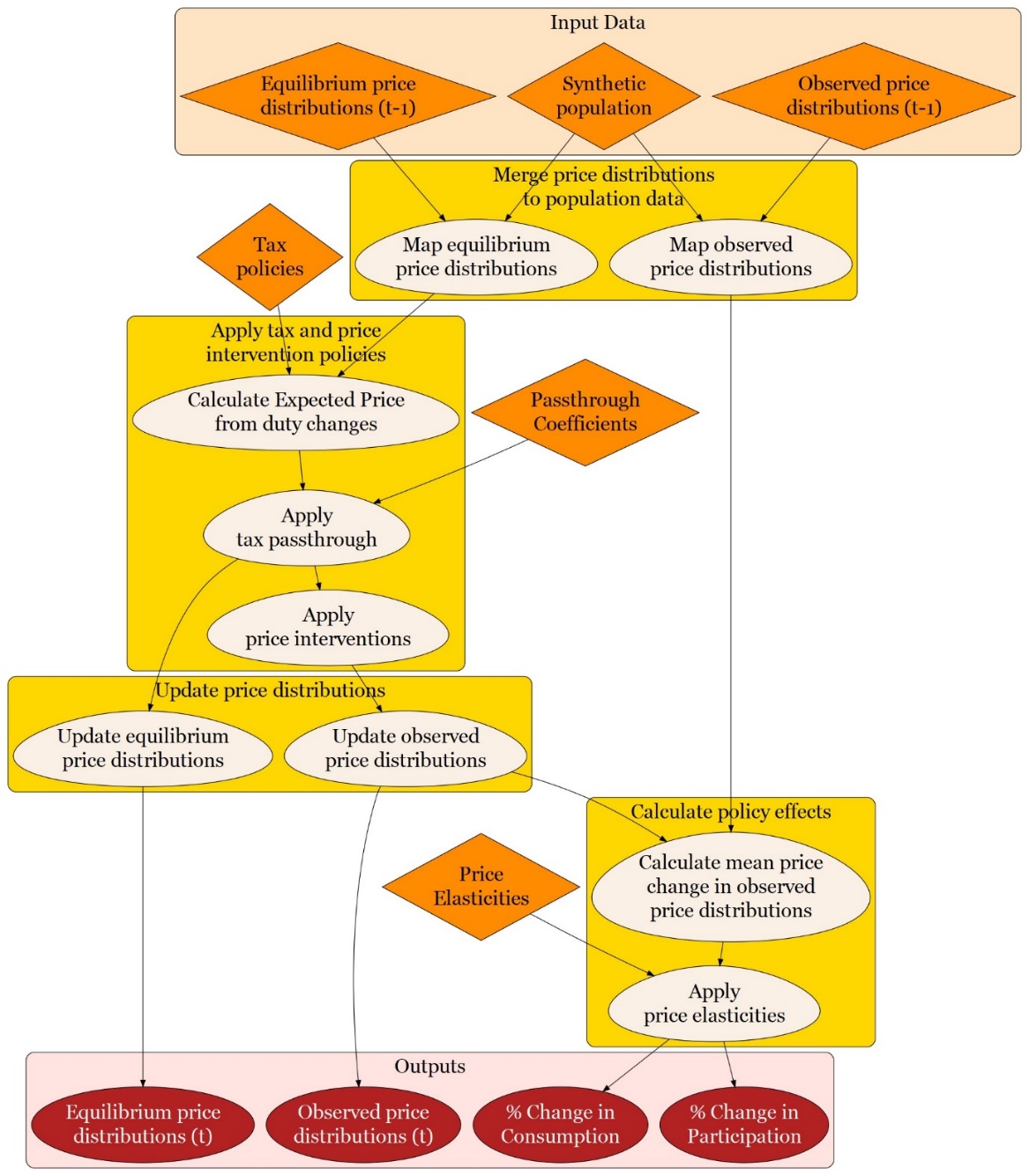


Figure 2 Summary of the TAX-Sim workflow for calculating price and consumption effects.

### Modelling tax policy to price

#### Price distributions

To model the effects of a tax change, it is necessary to assign individuals in the simulation a distribution of prices paid for tobacco and alcohol products. To do this, individuals are probabilistically assigned to a distribution of prices paid based on their age, sex, income, tobacco consumption and alcohol consumption. This is done so we can understand the mean price that each individual pays for a product, the tax breakdown of these prices and how the mean prices paid change due to a tax change.

The price distributions in the model are subgroup level and split by age group, sex, income quintile, drinker category, and smoker category. Price distributions are characterised as population subgroup **j**'s consumption of product **k** by dividing the distribution into 50 transaction price bands and recording the proportion of total spending on product **k** by subgroup **j** occurring at transaction price band **l**. For further detail on the construction of the price distributions from LCFS data (section ‎1.2.3.3).

All transactions in a price band are assumed to occur at the price representing the mid-point of the band i.e. purchases between 50 and 59p per unit of alcohol (where a unit of alcohol is defined as 10ml/8g of pure ethanol) are set at 55p. Alcohol price distributions are defined over the range 0 - 500p per unit in 10p intervals, and tobacco price distributions are defined over the range 0 - 100p per stick in 2p intervals. A stick is defined as one factory-made cigarette or 0.5g of hand-rolling tobacco.

The average price an individual pays for a particular product is summarised as the mean of the price distribution they face, based on their subgroup. This is calculated as the mean value of all price points weighted by the share, $\boldsymbol{\delta}_{\boldsymbol{jkl}}$, of total consumption of product **k** by subgroup **j** that is purchased at price point **l**:

$$\bar{p}_{ijk}=\bar{p}_{jk}=\sum_{l=1}^{50} \delta_{jkl}p_{jkl}$$

The price distributions are merged into the simulated dataset in two steps:

(1) Since income quintile is not in the starting population, nor do we explicitly model the transition of income over time, the first step is to probabilistically assign individuals to one of 5 income quintiles based on their current age-band (18-24, 25- 34, 35-54, 55+), sex, IMDQ, tobacco consumption (5 levels) and alcohol consumption category (4 levels). The method to generate the probabilistic map between IMDQ and income quintiles uses 6 years' worth of data for HSE data from 2013 to 2018. This produces a look-up table that gives the probability an individual is in each of the 5 equivalised income quintile categories. In each year as the simulation progresses, the individual is Monte Carlo resampled as to their income quintile based on their updated characteristics.

(2) Merge the price distributions with the simulated individual dataset based on their simulated age, sex, income quintile and tobacco and alcohol consumption. Merging the price distribution with the simulated individual dataset in each year of the simulation ensures that the price distributions assigned to individuals in the microsimulation can vary over individual life courses, i.e., if an individual ages or changes their level of tobacco or alcohol consumption during the simulation.

Price distributions are expressed in real-terms values, adjusted for inflation by the Retail Price Index (RPI). All specific duty rates per unit are also expressed in real-terms, and so it is assumed that it is **real terms changes in duty and minimum price thresholds** that have an impact on the real price and so on consumption.

Prior to the policy effect year, the control and intervention arms of the model follow an identical time series of duty rates. In the policy effect year and subsequent years, the control arm assumes that alcohol duty is increased in line with inflation in nominal/cash terms (and so frozen in real terms). Tobacco duty is assumed to continue to increase in line with the duty escalator policy of RPI + 2%. This is assumed to be the case in all future years following the policy effect year.

In the intervention arm, the policy effect year is the year in which a policy change occurs e.g., a 10% increase in alcohol duty. An increase in duty of 10% is assumed to be over and above inflation, i.e., specified proportionate changes to duty rates are real terms increases. As in the control arm, alcohol duty is assumed to increase in line with inflation after the policy effect year and tobacco duty continues to increase in line with the escalator (though in the treatment arm the escalator can be adjusted, e.g., increased from 2% to 4% above RPI, or abolished entirely).

#### Calculate expected price

The first step in applying a tax policy change is to calculate the expected price, $\boldsymbol{p}^{\boldsymbol{EXP}}$, which results from a policy which changes specific alcohol duty or the rate of value-added tax. This is the price which would be set, with retailer net revenue held constant, under the new tax regime. It is calculated by decomposing retail price per unit into its tax and industry revenue components, adjusting the tax parameters to the new policy, and reconstructing price while holding industry revenue per unit constant.

The expected price calculated here assumes that the supply side of the alcohol/tobacco markets do not respond to changes in the levels of taxation. Assuming the new observed price to be the expected price calculated here does not allow for under-shifting (absorbing some of the tax increase) or over-shifting (increasing the price by more than needed to simply cover for increased tax burden). Expected prices are therefore further adjusted for tax passthrough, to obtain a new observed price following the tax policy change which reflects industry/retailer response to the tax changes.

#### Apply tax passthrough

Tax passthrough in the model is based on empirical estimates of industry behaviour. To estimate passthrough coefficients, quantile regressions are estimated at the mid-point of each decile of the observed price distribution, with observed price as the outcome variable and expected price as the independent variable. The tax passthrough coefficients are used to calculate the new observed price. The coefficients used represent the most up-to-date empirical estimates for off-trade alcohol^30^, on-trade alcohol^31^, and tobacco^32^.

### Modelling minimum unit pricing

A minimum unit price (MUP) is a legal minimum retail price below which a product cannot be sold to consumers, expressed in units of that product. In the context of alcohol this means a minimum price per unit of alcohol (defined as the UK standard unit of 10ml/8g of ethanol). The effects of MUP have previously been modelled using the Sheffield Alcohol Policy Model (SAPM)^33,34^, and the methods developed for the present STAPM analysis build on this work.

Minimum unit pricing is applied by constructing a parallel price distribution. The price distribution that has been described so far in this document is an equilibrium price distribution. This is a price distribution which reflects changes in taxation for alcohol and tobacco products, but not any direct price control policies such as MUP. A minimum unit price represents a state of dis-equilibrium for products which are affected by it, and it is assumed that if a minimum unit price were removed or reduced, prices affected by the MUP would adjust back towards their equilibrium values.

Each year of the simulation, the equilibrium price distribution at time ***t*** is updated following any changes to tax policy and saved. This is then used as the input price distribution in time ***t+1*** to which the next year tax policies will be applied. An observed price distribution is constructed for time ***t*** by applying the minimum unit price to the equilibrium price distribution.

The price paid by subgroup ***j*** for product ***k*** at price point ***l*** is set with reference to the equilibrium price distribution. The observed price distribution price, $p_{jkl}^{obs}$ is identical to the equilibrium distribution price $p_{jkl}^{eq}$ unless $p_{jkl}^{eq}$ is less than the minimum unit price for product ***k***, $p_{k}^{MUP}$, in which case the price is increased to the minimum price.

$$p_{jkl}^{obs}=max(p_{jkl}^{eq}, p_{k}^{MUP})$$

The observed price distributions in time ***t*** and ***t-1*** are used to calculate the change in mean prices. Note that with no minimum unit price ($p_{k}^{MUP}$= 0), the observed and equilibrium price distributions will be identical. The equilibrium price distribution is passed to the next time step of the model for calculating the impact of further changes to tax in the next period. This is because tax impacts on price affect the underlying equilibrium distribution before any impact of MUP is considered.

Without price controls (i.e. MUP) influencing the market, prices are assumed to be set such that profits are maximized, conditional on all other market conditions (i.e. costs of production, taxes). Industry will therefore aim to keep prices as close to the equilibrium price as possible, subject to the constraint of adhering to the MUP threshold, as this is by assumption the price at which profits are maximised. For example, if an observed price is 50p due to the presence of MUP, but in the absence of MUP would be 35p, the effect of a tax intervention which increased the equilibrium price from 35p to 40p would leave the observed price unchanged at 50p, as 50p is still the closest price possible to the profit maximising price.

The presence of an underlying equilibrium price distribution also allows for modelling of reductions in the minimum unit price. A MUP may be reduced over time for two reasons. Firstly, there may be a deliberate policy decision to reduce the MUP threshold. Secondly, because prices in the model are expressed in real terms, so is the minimum unit price. If the MUP threshold is not uprated over time (i.e. is kept constant in cash terms), the MUP in real terms will reduce and therefore become less effective over time because of inflation.

Figure A3 illustrates the modelling of reductions in MUP, using an example price distribution for off-trade beer. Assume prices remain constant in real terms and there are no tax changes in the time frame considered. In this case, the equilibrium price distribution will be identical to the solid blue line at each time point. If a MUP policy of 50p per unit is introduced in time $t_{1}$, the observed price distribution is truncated at 50p, with all prices below 50p being raised to the new MUP threshold.


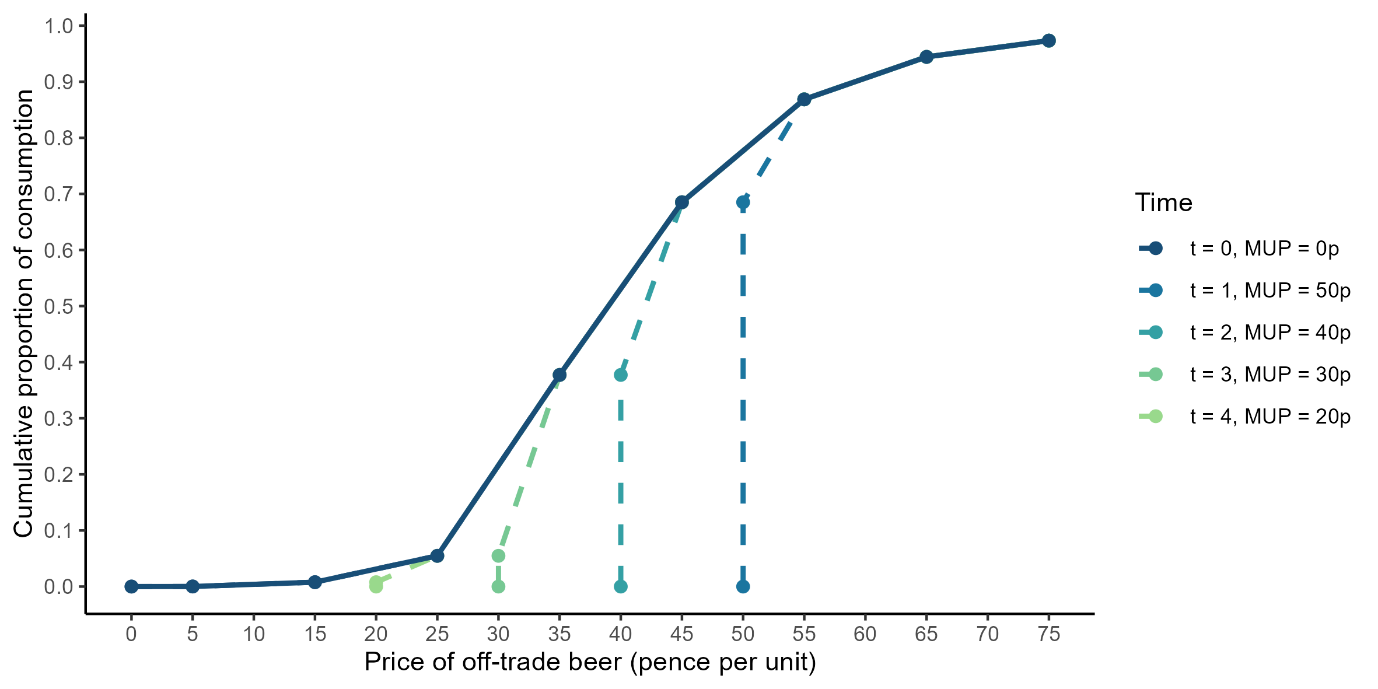


Figure 3 Illustration of the impact of a hypothetical MUP policy on the price distribution.

Assume that in subsequent periods MUP is held constant at 50p in cash terms, and the impact of inflation is to reduce the real terms MUP to 40p in $t_{2}$, 30p in $t_{3}$, and 20p in$t_{4}$. As Figure A3 shows, in time $t_{2}$ products priced at 45p in $t_{0}$, revert to that price, while products which were priced at 40p and below in $t_{0}$ fall from 50p to 40p. Over time, with no further adjustment to the cash value of the MUP threshold, the real terms observed price distribution converges back to the equilibrium price distribution as the MUP is eroded by inflation.

### Modelling price to consumption

In this section we describe how price changes resulting from tax and price policies, as described in the previous sections, translate into changes in consumption through price elasticities.

#### Price elasticities of demand

The price elasticities of demand model the effect on whether an individual consumes a product at all (participation), and for those that are consumers of a product, how much of it they consume (conditional consumption). Estimates of consumer responses to price changes take the form of 'price elasticities of demand', or the proportional change in consumption for a unit proportional change in price. The 'own-price' elasticity tells us the response of demand for a product to a change in its own price. The 'cross-price' elasticity tells us the response due to a change in another product's price.

There are two types of elasticity - participation and conditional consumption. Participation elasticities measure the percentage change in participation (the probability of an individual consuming, or the proportion of a subgroup who are consumers) for a percentage change in price. Conditional consumption elasticities measure the percentage change in the amount consumed by consumers for a percentage change in price. Elasticities are entered into the model as a set of square matrices (one for participation and one for conditional consumption) with 12 rows and columns to account for each of the 10 alcohol and 2 tobacco product own-price and cross-price elasticities which apply to all individuals in the population.

There is a substantial literature on the estimation of price elasticities for alcohol^35^ and for tobacco^36^ separately, but little evidence on joint estimation of tobacco and alcohol price elasticities. For the UK, there are no studies in which a single econometric modelling approach is used to estimate joint price elasticities for participation in, and consumption of, both tobacco and alcohol products simultaneously.

The matrix below represents a general price elasticity matrix with *n* products. Each element $\varepsilon_{x,y}$in the matrix represents the price elasticity of demand for product y with respect to the price of product x. On the lead diagonal x = y, and the elasticity is an own-price elasticity – the responsiveness of consumption of a product to changes in its own price. Off-diagonal elements are cross-price elasticities, e.g., $\varepsilon_{1,2}$is the elasticity for a change in consumption of product 2, given a 1% increase in the price of product 1.

$$\left( \begin{matrix} \varepsilon_{1,1} & \cdots& \varepsilon_{1,n} \\ \vdots& \ddots& \vdots\\ \varepsilon_{n,1} & \cdots& \varepsilon_{n,n} \end{matrix} \right)$$

As proportional changes to product price in the model are stratified by population subgroup, proportionate changes in participation and conditional consumption are calculated for each population subgroup. These proportionate changes are then applied to individual level consumption based on their subgroup.

The base case price elasticities, Pryce et al.^37^, used in the model were estimated using Living Costs and Food Survey (LCFS) data from 2006 – 2017. Using a generalised Tobit regression model, two 12 by 12 elasticity matrices were produced - one for participation elasticities and one for conditional consumption elasticities. For sensitivity analyses, the model can use other elasticities instead, i.e., produced by other methods or comparing to other elasticity estimates in the literature. The participation and conditional consumption elasticities used in the model are presented in Table A5 and Table A5. Note that while these tables report the elasticities as estimated, in the modelling the elasticities to/from tobacco are switched to zero to isolate the effects of changes in alcohol prices only. As a sensitivity analysis which can be used by the TAX-sim model we also applied the Meng et al (2014)^38^ (alcohol) price elasticities which are reported in Table A6.

One limitation to the use of elasticities in the TAX-sim model is that there is currently no capacity to investigate statistical uncertainty in the elasticity estimates. Each price elasticity has an associated standard error and confidence intervals reflecting the uncertainty in the empirical estimates. While structural sensitivity analysis can be conducted (by e.g., setting cross-price elasticities to zero, or setting statistically insignificant price elasticities to zero), a probabilistic sensitivity analysis exploring the impact of uncertainty in the estimates of the price elasticities is not possible.

Table A4 Base Case Pryce et al. elasticities (participation)

| / Quantity Price/ | Off Beer | Off Cider | Off Wine | Off Spirits | Off RTDs | On Beer | On Cider | On Wine | On Spirits | On RTDs | FM Cigs | RYO Tob |
| --- | --- | --- | --- | --- | --- | --- | --- | --- | --- | --- | --- | --- |
| Off Beer | -0.25 | -0.04 | 0.00 | -0.05 | -0.01 | -0.03 | -0.01 | 0.02 | -0.00 | -0.00 | -0.05 | -0.02 |
| Off Cider | -0.03 | -0.12 | -0.02 | -0.04 | -0.00 | 0.00 | -0.01 | 0.00 | 0.02 | -0.00 | -0.02 | -0.02 |
| Off Wine | -0.06 | -0.04 | -0.31 | -0.03 | -0.01 | -0.02 | -0.01 | 0.00 | -0.01 | -0.00 | -0.04 | -0.01 |
| Off Spirits | -0.06 | -0.04 | -0.05 | -0.20 | -0.02 | 0.00 | -0.00 | -0.01 | -0.02 | -0.01 | -0.03 | -0.02 |
| Off RTDs | 0.00 | 0.00 | -0.03 | -0.03 | -0.03 | 0.03 | -0.00 | -0.01 | 0.01 | 0.01 | 0.00 | 0.01 |
| On Beer | -0.04 | -0.00 | 0.05 | -0.01 | -0.00 | -0.29 | -0.02 | 0.09 | -0.01 | -0.01 | -0.09 | -0.03 |
| On Cider | 0.01 | -0.02 | 0.01 | -0.01 | 0.01 | -0.00 | -0.09 | 0.01 | -0.02 | -0.01 | -0.00 | 0.01 |
| On Wine | -0.02 | -0.01 | -0.07 | -0.02 | -0.01 | -0.12 | -0.02 | -0.24 | -0.01 | -0.01 | 0.00 | 0.01 |
| On Spirits | -0.03 | -0.01 | -0.01 | -0.03 | -0.00 | -0.12 | -0.03 | -0.00 | -0.18 | -0.03 | -0.02 | -0.02 |
| On RTDs | 0.00 | 0.00 | 0.01 | -0.02 | 0.01 | 0.01 | 0.01 | 0.03 | -0.01 | -0.01 | 0.02 | -0.01 |
| FM Cigs | 0.04 | -0.03 | 0.30 | 0.02 | -0.03 | 0.21 | 0.02 | 0.12 | 0.06 | -0.01 | -0.17 | -0.14 |
| RYO Tob | -0.04 | 0.02 | -0.01 | -0.05 | 0.01 | 0.05 | -0.01 | 0.02 | 0.01 | 0.00 | -0.08 | -0.09 |

Table A5 Base Case Pryce et al. elasticities (conditional consumption)

| / Quantity Price/ | Off Beer | Off Cider | Off Wine | Off Spirits | Off RTDs | On Beer | On Cider | On Wine | On Spirits | On RTDs | FM Cigs | RYO Tob |
| --- | --- | --- | --- | --- | --- | --- | --- | --- | --- | --- | --- | --- |
| Off Beer | -1.20 | -0.05 | -0.04 | -0.11 | -0.08 | -0.12 | -0.11 | -0.04 | -0.21 | -0.04 | -0.16 | -0.11 |
| Off Cider | -0.07 | -1.14 | -0.13 | -0.10 | 0.02 | -0.07 | -0.33 | 0.05 | 0.07 | 0.01 | 0.03 | -0.00 |
| Off Wine | -0.00 | 0.05 | -0.34 | -0.06 | 0.06 | 0.01 | 0.05 | 0.06 | 0.03 | 0.22 | -0.05 | 0.06 |
| Off Spirits | 0.02 | 0.02 | 0.01 | -0.22 | -0.07 | -0.04 | -0.08 | 0.06 | -0.06 | 0.00 | 0.02 | 0.02 |
| Off RTDs | 0.00 | -0.02 | -0.11 | 0.00 | -0.49 | -0.02 | 0.05 | -0.04 | -0.06 | -0.07 | 0.06 | 0.16 |
| On Beer | -0.05 | 0.04 | 0.08 | 0.05 | 0.01 | -0.80 | -0.13 | 0.15 | 0.02 | -0.15 | -0.18 | -0.11 |
| On Cider | 0.09 | -0.17 | -0.05 | 0.08 | 0.09 | -0.07 | -0.34 | 0.01 | 0.04 | 0.03 | 0.00 | 0.31 |
| On Wine | 0.07 | 0.04 | -0.04 | -0.00 | 0.19 | -0.04 | 0.03 | -0.39 | 0.06 | -0.07 | 0.03 | 0.01 |
| On Spirits | -0.01 | -0.03 | 0.06 | 0.01 | -0.02 | -0.18 | -0.17 | -0.01 | -0.78 | -0.06 | -0.01 | 0.14 |
| On RTDs | -0.02 | -0.08 | -0.03 | -0.00 | -0.10 | -0.02 | 0.11 | 0.05 | 0.03 | -0.14 | -0.03 | 0.00 |
| FM Cigs | -0.29 | -1.02 | 0.40 | 0.36 | 0.36 | 0.45 | -0.23 | 0.40 | 0.38 | -0.50 | -0.51 | -0.22 |
| RYO Tob | -0.10 | 0.04 | -0.01 | 0.01 | -0.34 | 0.00 | -0.43 | 0.12 | -0.02 | 0.28 | -0.08 | -0.23 |

Table A6 Meng et al. (2014) alcohol price elasticities

| / Quantity Price/ | Off Beer | Off Cider | Off Wine | Off Spirits | Off RTDs | On Beer | On Cider | On Wine | On Spirits | On RTDs | FM Cigs | RYO Tob |
| --- | --- | --- | --- | --- | --- | --- | --- | --- | --- | --- | --- | --- |
| Off Beer | -0.98 | -0.19 | 0.10 | -0.37 | -1.09 | -0.02 | -0.05 | 0.25 | 0.03 | 0.50 | 0.00 | 0.00 |
| Off Cider | 0.07 | -1.27 | 0.12 | -0.12 | -0.24 | -0.05 | 0.09 | 0.07 | -0.11 | -0.19 | 0.00 | 0.00 |
| Off Wine | -0.04 | 0.74 | -0.38 | 0.36 | 0.04 | -0.25 | -0.16 | 0.04 | -0.19 | 0.11 | 0.00 | 0.00 |
| Off Spirits | 0.11 | -0.02 | 0.16 | -0.08 | -0.04 | 0.17 | 0.41 | 0.01 | 0.08 | 0.23 | 0.00 | 0.00 |
| Off RTDs | -0.05 | -0.16 | -0.01 | 0.08 | -0.59 | -0.06 | 0.07 | 0.07 | 1.18 | 0.09 | 0.00 | 0.00 |
| On Beer | 0.15 | -0.29 | 0.12 | -0.03 | 0.80 | -0.79 | 0.87 | 1.04 | 1.17 | -0.12 | 0.00 | 0.00 |
| On Cider | -0.10 | 0.07 | 0.04 | 0.02 | 0.37 | 0.04 | -0.59 | 0.07 | 0.24 | 0.24 | 0.00 | 0.00 |
| On Wine | -0.20 | 0.09 | -0.15 | -0.03 | -0.09 | -0.28 | -0.03 | -0.87 | -0.02 | -0.36 | 0.00 | 0.00 |
| On Spirits | 0.02 | -0.12 | -0.03 | -0.28 | -0.15 | 0.00 | -0.28 | 0.11 | -0.89 | 0.81 | 0.00 | 0.00 |
| On RTDs | 0.08 | 0.01 | -0.09 | -0.05 | 0.37 | 0.12 | -0.39 | -0.03 | -0.07 | -0.19 | 0.00 | 0.00 |
| FM Cigs | 0.00 | 0.00 | 0.00 | 0.00 | 0.00 | 0.00 | 0.00 | 0.00 | 0.00 | 0.00 | 0.00 | 0.00 |
| RYO Tob | 0.00 | 0.00 | 0.00 | 0.00 | 0.00 | 0.00 | 0.00 | 0.00 | 0.00 | 0.00 | 0.00 | 0.00 |

### Updating consumption

Updating consumption involves first updating the participation of individuals in consumption of products, and then the amount consumed by individuals who remain consumers after the participation decision is made.

#### Adjusting participation

Firstly, the number of consumers who will switch consumption is calculated on a product-by-product basis. The prevalence within each subgroup **j** of consumption of product **k** prior to the price change - $\boldsymbol{\rho}_{\boldsymbol{jk,t-1}}$- is calculated and matched with the relative changes calculated from the participation elasticities, $\boldsymbol{\%\Delta}\boldsymbol{p}_{\boldsymbol{jk,t}}$. The new prevalence following the price change is then:

$$\rho_{jk,t}=\rho_{jk,t-1}(1+\boldsymbol{\%\Delta}\boldsymbol{p}_{\boldsymbol{jk,t}})$$

The change in the number of consumers by subgroup, $N_{j}^{(c)},$ is then simply the size of the subgroup, $N_{j}$, multiplied by the change in prevalence:

$$\Delta N_{j}^{\left( c \right)}=N_{j}\left( \rho_{jk,t}-\rho_{jk,t-1} \right)$$

If the number of consumers is falling, then individuals in the subgroup are chosen at random to become non-consumers and have their consumption adjusted to zero. If the number of consumers who will switch is greater than the number of current consumers, then all consumers in the subgroup have their consumption switched to zero.

Conversely, if the number of consumers is rising, then non-consumers in the subgroup are chosen at random to become consumers. If the number of consumers who will switch is greater than the number of current non-consumers, then all remaining non-consumers in the subgroup will become consumers. Non-consumers are chosen at random to become consumers and they are assigned a level of consumption and associated product preferences by taking a random sample of the consumption of current consumers in the model within their subgroup.

#### Adjusting conditional consumption

With participation adjusted, the level of consumption is next adjusted to reflect changes to the amount of product consumed conditional on the individual being a consumer. As with participation, the new consumption is calculated by combining the initial (individual level) consumption with the relative changes at subgroup level calculated from the conditional consumption elasticities.

$$c_{jk,t}=c_{jk,t-1}(1+\%\Delta c_{jk,t})$$

Note that individual subgroups are determined partly by level of alcohol and tobacco consumption. These subgroups change dynamically such that if, for example, the combined policy effects of a duty change result in a drinkers' mean weekly consumption reducing from the level of a harmful drinker to that of a hazardous drinker, their drinker category is updated to reflect the change in consumption. This represents a change from the SAPM approach, in which an individual's drinker category is static and defined by drinking at baseline. Comparisons of outcomes within drinker and/or smoking categories across different models should therefore be treated with caution, as the composition of those groups will not be identical even given the same initial synthetic population.

### Calculating economic outcomes

#### Calculating total spending, tax receipts, and industry revenue

Total spending and the division of total spending into tax revenues and retail/industry revenues are calculated by matching individual level consumption data to subgroup-level mean prices and tax payment data saved from the TAX-sim simulation run. Mean price, consumption, duty, and VAT are then calculated, stratified by product, year, and arm of the model. This calculation is weighted by the number of individuals in the total population that each individual in the synthetic population represents. The calculation can additionally be stratified by any subgroup defined by variables in the consumption data e.g., by Index of Multiple Deprivation quintile.

Total consumption is calculated by multiplying the weighted mean of consumption by the total number of people in the population. Prior to this calculation, all consumption is adjusted to weekly figures (multiplying daily consumption of cigarettes and hand-rolled tobacco by 7). The total spending is then calculated as this total consumption figure multiplied by the mean price. Total VAT and total duty are similarly calculated as their respective subgroup-level means multiplied by total consumption in that subgroup. Total industry/retail revenue is then calculated as a residual - total expenditure minus total tax receipts. In addition to a mean price figure, a mean "basic price" figure is calculated, which is the retail price minus the tax components (equivalent to the net retail/industry revenue per unit of consumption). The weekly spending, tax, and revenue figures are then annualised by multiplying through by 52 weeks per year.

These calculations are performed for each of the 12 products in the model. The number of products can be aggregated. The two tobacco products can be left separated or combined into an all-tobacco figure. There are a number of ways the alcohol figures can be aggregated over products; an all-alcohol figure similar to tobacco, a 2-product categorisation which aggregates all figures by on-trade or off-trade channel of sale, and a 5-product categorisation where figures are produced for beer, cider, wine, spirits, and RTDs separately and aggregated over channel of sale. Aggregation is performed by summing up the total tax, revenue, and spending figures, and as a mean weighted by consumption in the case of prices.

#### Upshifting

Upshifting is performed to account for under-reporting of alcohol consumption/spending in survey data. In the default configuration of the TAX-sim model, the model projects alcohol consumption and estimates the associated health outcomes based on **reported** consumption (i.e. consumption that might be subject to under-reporting). However, in that default configuration, the estimates of total spending on alcohol, tax receipts, and industry revenues from alcohol sales have an adjustment applied to them to avoid underestimates in these outcomes due to the under-reporting of alcohol consumption. Thus, in the default model configuration, adjusted consumption figures are used to calculate more accurate figures for the economic outcomes, but the reporting of alcohol consumption and associated health outcomes is based on unadjusted consumption.

Upshifting of total spending on alcohol, tax receipts, and industry revenues from alcohol sales is performed by comparing the total duty receipts from alcohol estimated by the model with the actual total duty receipts reported by HM Revenue and Customs in a given reference year (by default, the reference year used is the year in which the model is initialised, but any year which is both included in the model and for which data from HMRC exists can be used). The data on actual duty receipts are obtained from the [alcohol bulletin](https://www.gov.uk/government/statistics/alcohol-bulletin), currently available up to the 2023/24 financial year. In order to obtain figures relevant for the country within the UK to which the TAX-sim model is parameterised for, the total UK duty receipts from alcohol are disaggregated into country-specific estimates using an [estimate of the country splits in duty receipts](https://www.gov.uk/government/statistics/disaggregation-of-hmrc-tax-receipts) derived from an analysis of the Living Costs and Food Survey (LCFS) data.

Alcohol duty receipts are reported separately for beer, cider, wine, and spirits (alcopops are grouped with spirits) with no differentiation between on-trade and off-trade alcohol sales. Duty receipts estimated by the model are aggregated to match these four beverage categories and the upshift factor to be applied to the economic outcomes is calculated as the ratio of HMRC reported duty receipts to the un-adjusted duty receipts estimated by the model for each of the four beverage categories.

The four upshift factors are then used in all calculations of economic outputs from the model. Each of the 10 products in the model are assigned the upshift factor for the relevant 4-category disaggregation of the upshift factor. For example, the consumption of off-trade wine and on-trade wine are multiplied by the same factor. The upshifting to obtain adjusted economic outcomes is applied to the unadjusted estimates of individual-level consumption and this adjusted alcohol consumption is then multiplied by prices of products purchased to obtain the adjusted estimates of total spending on alcohol, tax receipts, and industry revenues from alcohol sales.

**References**

1. Morris D, Brennan A, Angus C, Wilson LB, Pryce R, Gillespie D. Tobacco and Alcohol Tax and Price Intervention Simulation Model (TAX-sim): full technical documentation. *The University of Sheffield URL: https://osf.io/nfa4v* 2023.

2. Gillespie D, Morris D, Leeming G, et al. The Sheffield Alcohol Policy Model - new version coded in R (SAPM-R): full technical documentation. <https://osf.io/jkpx4>: The University of Sheffield, 2023.

3. Gillespie D, Brennan A. The Sheffield Tobacco Policy Model (STPM): full technical documentation. Version 2.0.1. <https://osf.io/96e5b>: The University of Sheffield, 2023.

4. Ministry of Housing Communities & Local Government. English indices of deprivation 20192019. <https://www.gov.uk/government/statistics/english-indices-of-deprivation-2019> (accessed.

5. Meier P, Purshouse RC, Brennan A. Policy options for alcohol price regulation: the importance of modelling population heterogeneity. *Addiction* 2010; **105**(3): 383-93.

6. Public Health Scotland. MESAS monitoring report2022. <https://www.publichealthscotland.scot/publications/mesas-monitoring-report-2022/> (accessed Sept 09 2024).

7. Brennan A, Meier P, Purshouse R, et al. The Sheffield Alcohol Policy Model - A Mathematical Description. *Health Economics* 2015; **24**(10): 1368-88.

8. Hill-McManus D, Angus C, Meng Y, Holmes J, Brennan A, Sylvia Meier P. Estimation of usual occasion-based individual drinking patterns using diary survey data. *Drug and Alcohol Dependence* 2014; **134**(C): 136-43.

9. Hill-Mcmanus D, Angus C, Meng Y, Holmes J, Brennan A, Meier P. Injury Alcohol-Attributable Fractions: Methodological Issues and Developments. Sheffield: University of Sheffield, 2014.

10. Holmes J, Meier PS, Booth A, Guo Y, Brennan A. The temporal relationship between per capita alcohol consumption and harm: A systematic review of time lag specifications in aggregate time series analyses. *Drug and Alcohol Dependence* 2012; **123**(1-3): 7-14.

11. Gillespie D, Webster L, Leeming G, Morris D, Angus C, Brennan A. hseclean: An R Package for Health Survey Data Wrangling. 1.11.3 ed: University of Sheffield; 2023.

12. Stockwell T, Donath S, Cooper-Stanbury M, Chikritzhs T, Catalano P, Mateo C. Under-reporting of alcohol consumption in household surveys: a comparison of quantity-frequency, graduated-frequency and recent recall. *Addiction* 2004; **99**(8): 1024-33.

13. Meng Y, Holmes J, Hill-McManus D, Brennan A, Meier PS. Trend analysis and modelling of gender-specific age, period and birth cohort effects on alcohol abstention and consumption level for drinkers in Great Britain using the General Lifestyle Survey 1984-2009. *Addiction* 2014; **109**(2): 206-15.

14. Angus C, Holmes J, Pryce R, Meier P, Brennan A. Alcohol and cancer trends: Intervention scenarios. Projecting Trends In Alcohol Consumption And Alcohol-Related Harm In England From 2015 To 2035 And Estimating The Impact Of Potential Minimum Unit Pricing And Taxation Policies Using The Sheffield Alcohol Policy Model Version 3.1: University of Sheffield and Cancer Research UK, 2016.

15. Coulson T, Tuljarukar S. The dynamics of a quantitative trait in age-structured population living in a variable environment. *The American Naturalist* 2008; **172**(5): 599.

16. Lee RD, Carter LR. Modeling and forecasting US mortality. *Journal of the American statistical association* 1992; **87**(419): 659-71.

17. Angus C, Henney M, Webster L, Gillespie D. Alcohol-attributable diseases and dose-response curves for the Sheffield Alcohol Policy Model version 4.0, 2018.

18. Webster L, Angus C, Brennan A, Gillespie D. Smoking and the risks of adult diseases. 2018.

19. Prabhu A, Obi KO, Rubenstein JH. The Synergistic Effects of Alcohol and Tobacco Consumption on the Risk of Esophageal Squamous Cell Carcinoma: A Meta-Analysis. *American Journal of Gastroenterology* 2014; **109**(6): 821-7.

20. Hashibe M, Brennan P, Chuang SC, et al. Interaction between Tobacco and Alcohol Use and the Risk of Head and Neck Cancer: Pooled Analysis in the International Head and Neck Cancer Epidemiology Consortium. *Cancer Epidemiology Biomarkers & Prevention* 2009; **18**(2): 541-50.

21. Purshouse R, Brennan A, Latimer N, et al. Modelling to assess the effectiveness and cost-effectiveness of public health related strategies and interventions to reduce alcohol attributable harm in England using the Sheffield Alcohol Policy Model version 2.0. 2009.

22. Watson PE, Watson ID, Batt RD. Prediction of Blood-Alcohol Concentrations in Human-Subjects - Updating the Widmark Equation. *Journal of Studies on Alcohol* 1981; **42**(7): 547-56.

23. Posey D, Mozayani A. The estimation of blood alcohol concentration. *Forensic Science, Medicine, and Pathology* 2007; **3**(1): 33-9.

24. Cherpitel CJ, Ye Y, Bond J, Borges G, Monteiro MJA. Relative risk of injury from acute alcohol consumption: modeling the dose–response relationship in emergency department data from 18 countries. 2015; **110**(2): 279-88.

25. Kontis V, Mathers CD, Rehm J, et al. Contribution of six risk factors to achieving the 25x25 non-communicable disease mortality reduction target: a modelling study. *Lancet* 2014.

26. Oza S, Thun MJ, Henley SJ, Lopez AD, Ezzati M. How many deaths are attributable to smoking in the United States? Comparison of methods for estimating smoking-attributable mortality when smoking prevalence changes. *Preventive medicine* 2011; **52**(6): 428-33.

27. Mansournia MA, Altman DG. Population attributable fraction. *Bmj* 2018; **360**.

28. Rosen L. An intuitive approach to understanding the attributable fraction of disease due to a risk factor: the case of smoking. *International Journal of Environmental Research and Public Health* 2013; **10**(7): 2932-43.

29. Gunning-Schepers L. The health benefits of prevention: a simulation approach. *Health Policy* 1989; **12**(1-2): 1-255.

30. Ally AK, Meng Y, Chakraborty R, et al. Alcohol tax pass-through across the product and price range: do retailers treat cheap alcohol differently? *Addiction* 2014; **109**: 1994-2002.

31. Wilson LB, Pryce R, Angus C, Hiscock R, Brennan A, Gillespie D. The effect of alcohol tax changes on retail prices: how do on-trade alcohol retailers pass through tax changes to consumers? *The European Journal of Health Economics* 2021; **22**(3): 381-92.

32. Wilson LB, Pryce R, Hiscock R, Angus C, Brennan A, Gillespie D. Quantile regression of tobacco tax pass-through in the UK 2013--2019. How have manufacturers passed through tax changes for different tobacco products? Tobacco Control: BMJ Publishing Group Ltd; 2020.

33. Holmes J, Meng Y, Meier PS, et al. Effects of minimum unit pricing for alcohol on different income and socioeconomic groups: a modelling study. *The Lancet* 2014; **383**(9929): 1655-64.

34. Meier PS, Holmes J, Angus C, Ally AK, Meng Y, Brennan A. Estimated Effects of Different Alcohol Taxation and Price Policies on Health Inequalities: A Mathematical Modelling Study. *PLOS Medicine* 2016; **13**(2): e1001963-e.

35. Wagenaar AC, Salois MJ, Komro KA. Effects of beverage alcohol price and tax levels on drinking: a meta‐analysis of 1003 estimates from 112 studies. *J Addiction* 2009; **104**(2): 179-90.

36. Gallet CA, List JA. Cigarette demand: a meta‐analysis of elasticities. *Health economics* 2003; **12**(10): 821-35.

37. Pryce R, Wilson LB, Gillespie D, Angus C, Morris D, Brennan A. Estimation of integrated price elasticities for alcohol and tobacco in the United Kingdom using the living costs and food survey 2006–2017. *Drug and Alcohol Review* 2023.

38. Meng Y, Brennan A, Purshouse R, et al. Estimation of own and cross price elasticities of alcohol demand in the UK—A pseudo-panel approach using the Living Costs and Food Survey 2001–2009. *Journal of Health Economics* 2014; **34**(C): 96-103.
